# Inhibition of HSP 90 is associated with potent anti-tumor activity in Papillary Renal Cell Carcinoma

**DOI:** 10.1186/s13046-022-02416-z

**Published:** 2022-06-27

**Authors:** Roma Pahwa, Janhavi Dubhashi, Anand Singh, Parthav Jailwala, Alexei Lobanov, Craig J. Thomas, Michele Ceribelli, Kelli Wilson, Christopher J. Ricketts, Cathy D. Vocke, Catherine Wells, Donald P. Bottaro, W. Marston Linehan, Len Neckers, Ramaprasad Srinivasan

**Affiliations:** 1 Urologic Oncology Branch, Center for Cancer Research, National Cancer Institute, National Institutes of Health, Bethesda, MD 20892 USA; 2 Present Address: Emory University School of Medicine, Atlanta, GA USA; 3 Thoracic Surgery Branch, National Cancer Institute, NIH, CCR and The Clinical Center, Bethesda, MD USA; 4 CCR Collaborative Bioinformatics Resource (CCBR), Frederick National Laboratory for Cancer Research, Leidos Biomedical Research, Inc, Frederick, MD USA; 5 Division of Pre-Clinical Innovation, National Center for Advancing Translational Sciences (NCATS), NIH, Bethesda, MD USA

**Keywords:** High throughput screening, RNA Sequencing, HSP90, Papillary Kidney Cancer, Treatment, PI3K/AKT pathway, MEK/ERK1/2 pathway, E2F/MYC

## Abstract

**Background:**

There is no universally accepted treatment for patients with advanced papillary renal cell carcinoma (PRCC). The presence of activating mutations in *MET*, as well as gain of chromosome 7, where the *MET* gene is located, are the most common genetic alterations associated with PRCC, leading to the clinical evaluation of MET tyrosine kinase inhibitors (TKIs) in this cancer. However, TKIs targeting MET selectively, as well as multitargeted TKIs with activity against MET demonstrate modest efficacy in PRCC and primary and secondary treatment failure is common; other approaches are urgently needed to improve outcomes in these patients.

**Methods:**

High throughput screening with small molecule libraries identified HSP90 inhibitors as agents of interest based on antitumor activity against patient derived PRCC cell lines. We investigated the activity of the orally available HSP90 inhibitor, SNX2112 in vitro*,* using 2D/3D PRCC cell culture models and in vivo*,* in mice tumor xenograft models. The molecular pathways mediating antitumor activity of SNX2112 were assessed by Western blot analysis, Flow cytometry, RNA-seq analysis, Real Time qPCR and imaging approaches.

**Results:**

SNX2112 significantly inhibited cellular proliferation, induced G2/M cell cycle arrest and apoptosis in PRCC lines overexpressing MET*.* In contrast to TKIs targeting MET, SNX2112 inhibited both MET and known downstream mediators of MET activity (AKT, pAKT1/2 and pERK1/2) in PRCC cell lines. RNAi silencing of AKT1/2 or ERK1/2 expression significantly inhibited growth in PRCC cells. Furthermore, SNX2112 inhibited a unique set of E2F and MYC targets and G2M-associated genes. Interestingly, interrogation of the TCGA papillary RCC cohort revealed that these genes were overexpressed in PRCC and portend a poor prognosis. Finally, SNX-2112 demonstrated strong antitumor activity in vivo and prolonged survival of mice bearing human PRCC xenograft.

**Conclusions:**

These results demonstrate that HSP90 inhibition is associated with potent activity in PRCC, and implicate the PI3K/AKT and MEK/ERK1/2 pathways as important mediators of tumorigenesis. These data also provide the impetus for further clinical evaluation of HSP90, AKT, MEK or E2F pathway inhibitors in PRCC.

**Graphical Abstract:**

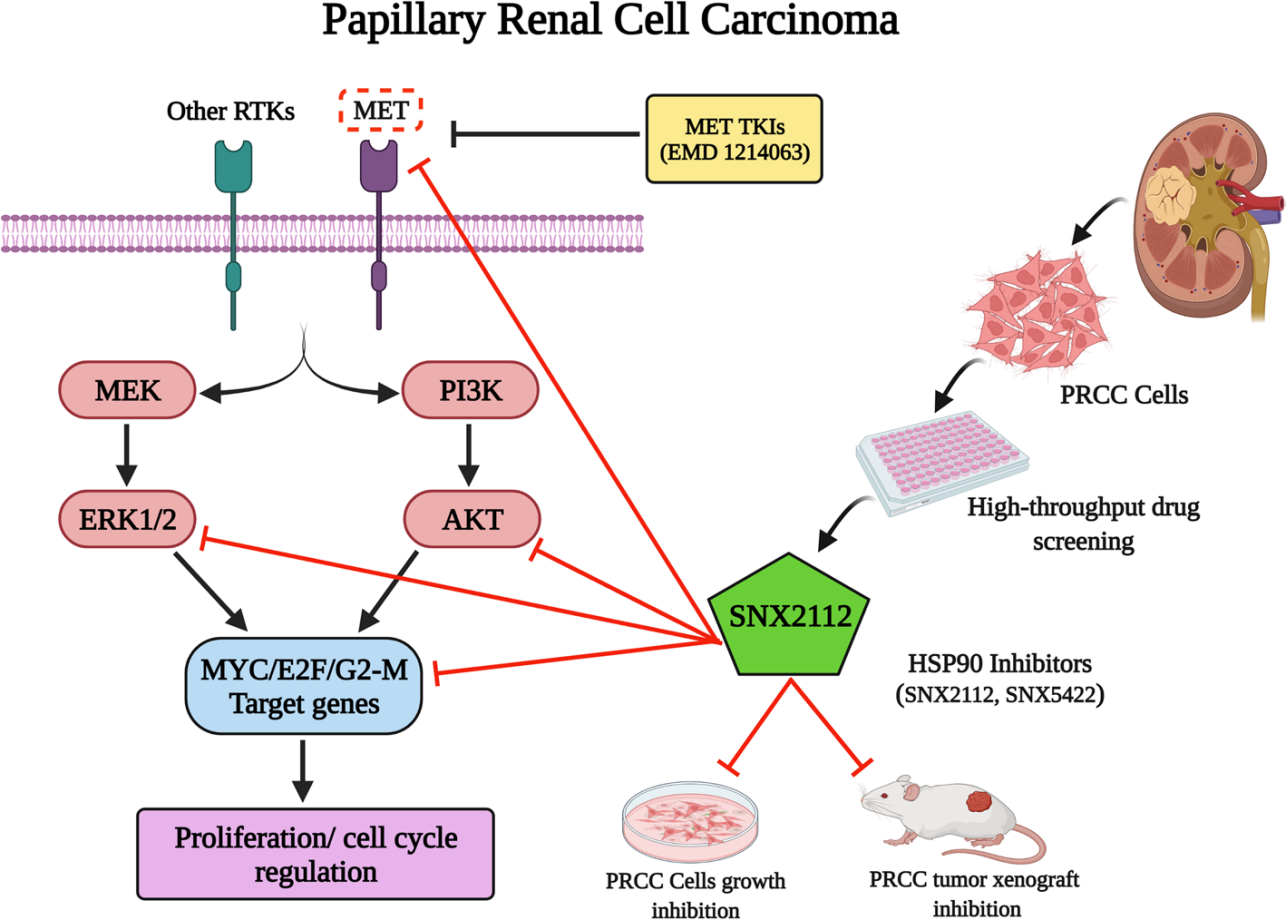

**Supplementary Information:**

The online version contains supplementary material available at 10.1186/s13046-022-02416-z.

## Introduction

Kidney cancer encompasses several histologically diverse cancers that arise in the kidney but are characterized by distinct clinical, histological, genetic and molecular features [1]. It was estimated that there were over 403,262 new cases of kidney cancer worldwide in 2018 and over 175,098 deaths [2]. The number of new kidney cancer cases per year is projected to increase further to 475,400 between 2018 and 2030 [3].

Papillary renal cell carcinoma (PRCC) is the second most common type of RCC after clear cell RCC, accounting for 15–20% of all kidney cancers [1]. PRCC can occur in both sporadic and hereditary forms, and based on histopathologic features, is subclassified into type 1 and type 2 variants [1, 4, 5]. However, there appears to be further heterogeneity at the molecular level as demonstrated by a comprehensive genomic characterization of 161 PRCC tumors by the Cancer Genome Atlas (TCGA) project that identified at least three distinct molecular signatures within the type 2 subgroup that segregated with outcome [5].

Several distinct genetic alterations were identified in type 2 PRCC tumors (including *CDKN2A, SETD2, BAP1, PBRM1, TERT, NF2, FH,* and *NRF2-ARE* pathway genes), while the most common abnormalities identified in type 1 PRCC included activating mutations of the *MET* gene and gain of chromosomes 7 and 17 [5]. Germline alterations in *MET* that result in constitutive activation of the MET pathway, are also the hallmark of hereditary papillary renal cell carcinoma (HPRC), an inherited condition that is associated with an increased risk of bilateral, multifocal, type 1 PRCC tumors [6, 7]. Although sporadic forms of type 1 PRCC are histologically identical to those seen in HPRC patients, activating somatic alterations in the *MET* gene are seen in only a minority (~ 13%) of these tumors [7–9]. However, gain of chromosome 7, that encodes *MET*, was seen in a much larger proportion (70–80%), notably chromosome 7 also encodes the MET ligand, HGF, and other potential oncologic driver genes, such as *EGFR* [5]*.* The presence of activating mutations in *MET*, as well as gain of chromosome 7, indicate that MET may be a clinically meaningful target in patients with type 1 PRCC.

Although agents targeting the Vascular Endothelial Growth Factor Receptor (VEGFR) pathway and immune checkpoint inhibitors have some activity, there is no universally accepted standard for treatment of most patients with advanced PRCC. Patients with PRCC treated with VEGF inhibitors, such as sunitinib, generally have worse outcomes and shorter Progression Free Survival (PFS) ranging from 1.6–6.6 months, than those with clear cell RCC (9–12 months) [10–12]. Tyrosine kinase inhibitors (TKIs) targeting MET have been evaluated in multiple clinical trials, demonstrating modest efficacy, largely restricted to patients with activating mutations in the MET tyrosine kinase domain [10, 11, 13, 14]. A phase II trial of foretinib, the first in class MET TKI inhibitor, with additional activity against VEGFR-2, demonstrated an overall response rate (ORR) of only 13% and a median PFS of 9.3 months in 74 patients with PRCC. Notably, the presence of germline *MET* mutations tended to correlate best with patient response to the drug, with 50% (five of ten patients) achieving a partial response [10, 13]; these findings were echoed in another phase II study that evaluated the efficacy of crizotinib, a dual MET and ALK inhibitor, in 23 patients with PRCC [10, 14]. More recently, a phase 3 study of the highly selective MET-tyrosine kinase inhibitor, savolitinib failed to demonstrate a clinical benefit over sunitinib, a multi-targeted receptor tyrosine kinase inhibitor without activity against MET, in patients with MET-driven PRCC [15]. Cabozantinib, a multi tyrosine kinase inhibitor with activity against VEGFR, MET, AXL and other kinases was recently shown to be superior to sunitinib in patients with advanced PRCC in a randomized phase 2 study and is reasonable therapeutic choice in some patients; however, the agent was associated with a modest ORR (23%) and PFS (9 months) [16]. In the same study, crizotinib and savolitinib, failed to show any improvement over sunitinib, in agreement with additional clinical data described above [16]. These data suggest that while the MET pathway might be important in a subset of PRCC, other approaches are urgently needed to improve outcomes in these patients.

In order to identify effective alternative pharmacologic approaches, we performed high throughput screening (HTS) to evaluate ~ 2000 potential small molecule anti-cancer agents against a panel of well characterized patient derived type 1 PRCC cell lines developed in our laboratory. Based on the screen, HSP90 inhibitors were selected for further investigation as agents with potential activity in PRCC. Small-molecule HSP90 inhibitors dissociate HSP90 from its clients, leading to their destabilization and eventual degradation [17–20]. HSP90 inhibitors have been reported to destabilize MET and several MET-activated downstream signaling proteins, including AKT and RAF that are important for cell growth and survival [17–19]. Of note, HSP90 inhibitors have also been shown to overcome resistance to MET-TKIs in pre-clinical MET-driven cancer models like gastric and lung cancers [19, 20]. We hypothesized that targeting HSP90 would be associated with antitumor activity in PRCC and lead to the evaluation of alternative therapeutic options for these patients.

In the current study, we extensively evaluate the efficacy of HSP90 inhibitors in pre-clinical models of PRCC cell lines with *MET* activating mutations as well as those with copy number gain of wild type (WT) *MET*.

## Materials and methods

### Reagents

The HSP90 inhibitor SNX2112 (PF-04928473) and SNX5422, an oral prodrug of SNX2112 was provided by Esanex, Inc. MG132 (carbobenzoxy-Leu-Leu-leucinal; 26S proteasome inhibitor) and Cyclohexamide (eukaryotic protein synthesis inhibitor) were procured from Sigma Aldrich.

### Cell culture

PRCC cell lines UOK332, UOK337, and UOK342 were derived from ascites obtained from patients with metastatic sporadic type 1 PRCC at the time of diagnosis, whereas UOK345 was derived from pleural effusion obtained from a patient with HPRC patient with multifocal, bilateral kidney tumors and widespread metastasis [21]. UOK 275 was derived from a retroperitoneal mass in a patient with PRCC as described before [22]. The renal proximal tubule epithelial cell line (RPTEC) was purchased from ATCC (Manassas, VA, USA). UOK345, UOK342, UOK337, UOK332 and UOK275 cells were cultured in high glucose DMEM containing pyruvate, glutamine and supplemented with 10% FBS. RPTEC cells were cultured and maintained according to ATCC.

### High-throughput screening

Briefly, PRCC cells were seeded with a Multidrop Combi dispenser into 1536 well white polystyrene tissue-culture treated plates, at a density of 500 cells per well in 5-µL volume. 23 nL of Mechanism Interrogation PlatE (MIPE) 4.0 compounds were added to individual wells (11 dosing tested for each compound in separate wells) via a 1536 pin-tool as described before [23]. Plates were incubated for 48 h at standard incubator conditions, covered by a stainless steel gasketed lid to prevent evaporation. Cell viability was assessed 48 h after compound addition by adding to each well 3 ul of CellTiterGlo luminescent substrate (Promega, Madison, WI, USA). Luminescence intensity values were acquired as median relative luminescence units (RLU) on a ViewLux reader with a 2 s exposure time per plate (PerkinElmer, Waltham, MA, USA). Data were normalized to intraplate DMSO (100% viability) and 2.3 µM bortezomib (0% viability) controls and expressed as Z-transformed measures of area under the curve (Z-AUC).

### Cell viability

PRCC cells, were seeded into 96-well plates at a density of 2.5 X 10^3^–3.0 X10^3^ cells/well. After 24 h, the cells were treated with different concentrations of each inhibitor (targeting MET or HSP90) for up to 72 h. Experiments with the MET inhibitor were performed in the presence of 100 ng/ml HGF. Cellular viability was measured using a commercially available Cell-Titer Glo kit (Promega, Madison, WI, USA) following the manufacturer’s protocol. Luminescence was acquired in a Perkin Elmer Microplate Luminometer (Perkin Elmer*,* Bedford, USA). To calculate survival the percentage of relative luminescence units (RLU) of the treated versus untreated cells were used. Studies were performed in three independent cell preparations.

### Cell invasion

Invasion assays were performed using the xCELLigence Real-Time Cell Analyzer (RTCA) Dual Purpose system (ACEA Biosciences, Inc., San Diego, CA). Briefly, UOK345 and UOK342 cells were serum-starved overnight and the following day, 4.0 × 10^4^ cells/ well were seeded into the upper chamber of RTCA CIM-Plate 16 (pre-coated with fibronectin) in DMEM media containing the MET inhibitor (50 or 500 nM) or vehicle. In bottom chamber, DMEM with HGF (100 ng/mL) or without HGF (negative control) were added. Impedance change generated from treated PRCC cells relative to vehicle alone–treated cells was continually monitored every 15 min and recorded in real-time for 24 h and expressed as cell index (CI).

### Clonogenicity

For colony formation, PRCC cells were trypsinized and seeded (1,000–1,500 cells/well) in six-well plates. Next day, the cells were treated with different concentrations of HSP90 inhibitor or vehicle. Every 72 h, the cells were replenished with inhibitor prepared in fresh media for 10–12 days. The foci were stained with crystal violet (0.5%) and imaged with microscopy.

### Anchorage-independent growth

Soft agarose was used for 3D colony formation assay. Six-well plates were pre-coated with bottom layer of 0.5% (w/v) agarose prepared in DMEM media and allowed to set. Viable PRCC cells (5,000/well) were resuspended in a soft agarose medium, consisting of 0.4% (w/v) agarose (Sigma-Aldrich), 10% FBS, and DMEM media with different concentrations of HSP90 inhibitor or vehicle, before being seeded on top of 0.5% (w/v) agarose. Cultures were incubated for 3 weeks. Colonies were stained with crystal violet solution (0.05%) and counted under a microscope.

### Cell transfection

For cell transfection, PRCC cells were seeded in triplicate in 96-well plates at 3000 cells/well in antibiotic-free complete medium and were allowed to adhere for 24 h. Thereafter, siRNAs (SMARTpool: ON-TARGETplus siRNA) and appropriate negative controls (Dharmacon, Chicago, IL) were transfected into cell lines at a final concentration of 50 nM using Lipofectamine RNAiMAX according to the manufacturer’s protocols (Thermo Fisher Scientific Inc., MA, USA). For overexpression of AKT1/2 or ERK1/2, GFP-tagged AKT1 (RG220257), AKT2 (RG217733), ERK1 (RG210493) and ERK2 (RG204196) transcript cloned downstream to CMV promoter into the pCMV6-AC-GFP expression vector (Origene, MD, USA) were used. For the generation of AKT1/2 overexpressing PRCC cell lines, cells seeded into six well plates at 2 × 10^5^ cells/well were co-transfected with AKT1 and AKT2 expression plasmids (1:1) using Lipofectamine 2000 with Opti-MEM (Thermo Fisher Scientific). Cells stably expressing AKT1/2 were selected under Neomycin (G418). Similarly, ERK1/2 overexpressing PRCC cells were generated by co-transfection of ERK1 and ERK2 expression vector. Cell growth was determined using a Cell-Titer Glo assay or Colony foci formation 48 h and 72 h following transfection.

### Western blot

For western blotting, cells were lysed in radio-immunoprecipitation buffer (Thermo Fisher Scientific Inc., MA, USA) and protein concentration of cell lysates was determined using the bicinchoninic acid protein assay kit (Thermo Fisher Scientific Inc., MA, USA). Equal amounts of protein lysates (10–20 μg) were loaded in 4–20% polyacrylamide gels (Biorad, Hercules, CA, USA) for electrophoresis and transferred onto a polyvinylidene difluoride membrane (BioRad, Hercules, CA). After protein transfer the membranes were blocked with 5% nonfat dry milk for 1 h and incubated with primary antibodies overnight at 4 °C. The following primary antibodies were used: β-actin (#8457), MET (#8198), phospho-MET (Tyr1230/1234/1235) (#3126), AKT (#9272), Phospho-Akt (Ser473) (#4060), p44/42 MAPK (Erk1/2) (#9102), Phospho-p44/42 MAPK (Erk1/2) (Thr202/Tyr204) (#9101), PARP (#9542) all at 1:1000 dilution (Cell Signaling Technology, Danvers, MA, USA) After primary antibody incubation the membranes were washed with TBS-Tween, and incubated for 45 min at room temperature with horseradish peroxidase-linked anti-rabbit secondary antibodies (#7074) at 1:3000 dilution (Cell Signaling Technology, Danvers, MA, USA) and visualized using the ECL protein detection system (Biorad, Hercules, CA, USA).

### Cell cycle

Briefly, cells were seeded in 10 cm dishes at a density of 5 × 10^5^ cells per dish and incubated for 24 h, followed by the addition of HSP90 inhibitor (50 nM) or vehicle for 48 h. After 48 h, cells were harvested and fixed with 70% ethanol overnight at 20 °C, and then stained with FxCycle™ PI/RNase staining solution at room temperature according to the manufacturer's instructions (Thermo Fisher Scientific Inc., MA, USA) and finally analyzed with a BD FACSCalibur™ platform according to manufacturer’s recommendations (BD Biosciences).

### Apoptosis

Briefly, PRCC cells at a density of 2.0 × 10^5^ cells/ well were treated for 48 h with HSP90 inhibitor (50 nM) or vehicle in 6-well plates. After 48 h, adherent and floating cells were collected and stained with Annexin V-FITC and propidium iodide at room temperature using Annexin V FITC Apoptosis Detection Kit from Abcam (Cambridge, MA, USA) according to the manufacturer's instructions, and finally analyzed with a BD FACSCalibur™ flow cytometer (Becton Dickinson).

### RNA sequencing analysis

For RNA seq, UOK345 and UOK342 cells were treated with HSP90 inhibitor (50 nM) or vehicle in 6-well plates and RNA was isolated after 48 h of treatment. The quality of total RNA was evaluated using the Agilent RNA Screen 2200 Tape Station system (G2964AA) (Agilent, Palo Alto, CA, USA). Only samples with a 28S/18S ribosomal peak ratio of 1.8–2.0 and a RIN number > 8.0 were considered suitable for sequencing. RNA-seq libraries were prepared using Illumina® TruSeq mRNA Prep Kit RS-122–2101 and were sequenced using Illumina® HiSeq3000/4000 to obtain 76-bp paired-end reads. The sequencing depth for each sample was > 20 million reads. RNA-seq data were processed using the CCBR Pipeliner (https://github.com/CCBR/Pipeliner) as follows: first, reads were trimmed to remove low-quality bases and adapter sequences using Cutadapt v 1.16 (https://doi.org/10.14806/ej.17.1.200). FastQC (http://www.bioinformatics.babraham.ac.uk) package was used to perform quality control, and reads were checked for contamination with FastqScreen v 0.9.3 (https://doi.org/10.12688/f1000research.15931.2) and Kraken (https://doi.org/10.1186/gb-2014-15-3-r46). On the next step, reads were mapped to hg38 human genome using STAR v2.5.2b (https://doi.org/10.1093/bioinformatics/bts635) in 2-pass mode. Then, gene-level expression was quantified using RSEM v1.3.0 (https://doi.org/10.1093/bioinformatics/bts635), with counts normalized to library size as counts-per-million. Finally, limma-voom v3.34.5 (https://doi.org/10.1186/1471-2105-12-323) was used for quantile normalization and differential expression. PCA plots and heatmaps were constructed using ClustVis (https://doi.org/10.1093/nar/gkv468) web service. The gene set enrichment analysis (GSEA) was performed using the Molecular Signatures Database (MSigDB) hallmark gene set collection. The RNA-seq data is available at GEO (GSE180820).

### Quantitative Real-time Polymerase Chain Reaction (qRT-PCR)

Total RNA was isolated from PRCC cells and tumor xenografts after treatment with HSP90 inhibitor using the RNeasy mini kit (Qiagen, MD) in accordance with the manufacturer’s protocol and quantified using a NanoDrop 2000 UV–Vis Spectrophotometer (Thermo Fisher Scientific Inc., MA, USA). For mRNA analysis, 2 μg of total RNA was reverse transcribed using the High-Capacity cDNA Reverse Transcription Kit (Thermo Fisher Scientific Inc., MA, USA). RT-PCR amplification was performed using an ABI ViiA7 real-time PCR system (Thermo Fisher Scientific Inc., MA, USA) and TaqMan® Gene Expression Assays (Thermo Fisher Scientific Inc., MA). Gene expression was normalized to beta-actin and calculated using ViiA7 software as comparative CT (ΔΔCT) values. qRT-PCR primers for gene expressions were obtained from (Thermo Fisher Scientific Inc., MA) (Table S1). All qRT-PCR -based reactions were performed independently in triplicate.

### Mouse Xenograft study

UOK345 or/ UOK342 cells (5 × 10^6^) were injected subcutaneously into the right flank of 5–7 weeks old female NSG (NOD.Cg-PrkdcscidIl2rgtm1Wjl/SzJ) mice [24]. For efficacy studies, when xenografts reached an average volume of approximately 100 mm^3^ after 4 weeks of injection, mice were randomized and divided into two groups (*n* = 10 for each group, mice bearing UOK342 xenografts and, *n* = 8 for each group, mice bearing UOK345 tumor xenografts). For all animal studies SNX2112 was delivered via its prodrug, SNX5422, that is orally bioavailable [19, 25, 26]. One group of UOK342 and UOK345 mice xenografts were treated with 30 mg/kg of SNX5422, (3 times per week on Mon, Wed and Fri schedule until 5 weeks via oral gavage) while the control groups received oral vehicle alone (1% carboxymethylcellulose/0.5% Tween 80) in a similar manner [19, 25, 26]. Tumor dimensions were measured with vernier calipers and tumor volumes were calculated using the formula V = 1/2 (length x width^2^) [27]. After 5 weeks, mice were sacrificed, tumors were excised, and tissues were snap frozen for signaling studies or fixed in 10% formalin and embedded in paraffin for additional pathologic studies. All animal experiments were done in compliance with our Animal Care and Use Committee in accordance with NIH Guidelines.

### Immunohistofluorescence

Tissue sections of tumor xenografts (5 µm) were deparaffinized and rehydrated serially in 100%, 95%, 70% and 50% ethanol, each for 5 min. According to manufacturer’s instructions, antigen retrieval was done using heated citrate buffer (Vector Laboratories, H3300). Expression of Ki-67 protein was monitored using a standard immunolabeling protocol with specific antibodies as follows: primary antibody against Ki-67 (#9027, Cell Signaling Technology) and secondary antibody Alexafluor488-conjugated, goat anti-rabbit (111–545-144, Jackson ImmunoResearch). Finally, medium containing DAPI (Vector Laboratories) was used for mounting tissue sections, and imaging was performed using a Zeiss LSM 710 NLO confocal microscope.

### Statistics

Fold or percentage change show the differences between experimental and control samples. Two-tailed, unpaired Student’s t test was used to calculate the significance between two conditions. Bar and line graphs were used to depict the mean ± standard error of the mean (SEM) or ± standard deviation (SD) as indicated for specific experimental runs of triplicate experiments. GraphPad Prism version (v.) 7.01 software was used for statistical calculations. Kaplan–Meier and log rank tests were used for overall survival analysis. *P* < 0.05 was considered significant.

## Results

### Overexpression of MET in patient derived papillary renal cancer cell lines

**Fig. 1:**
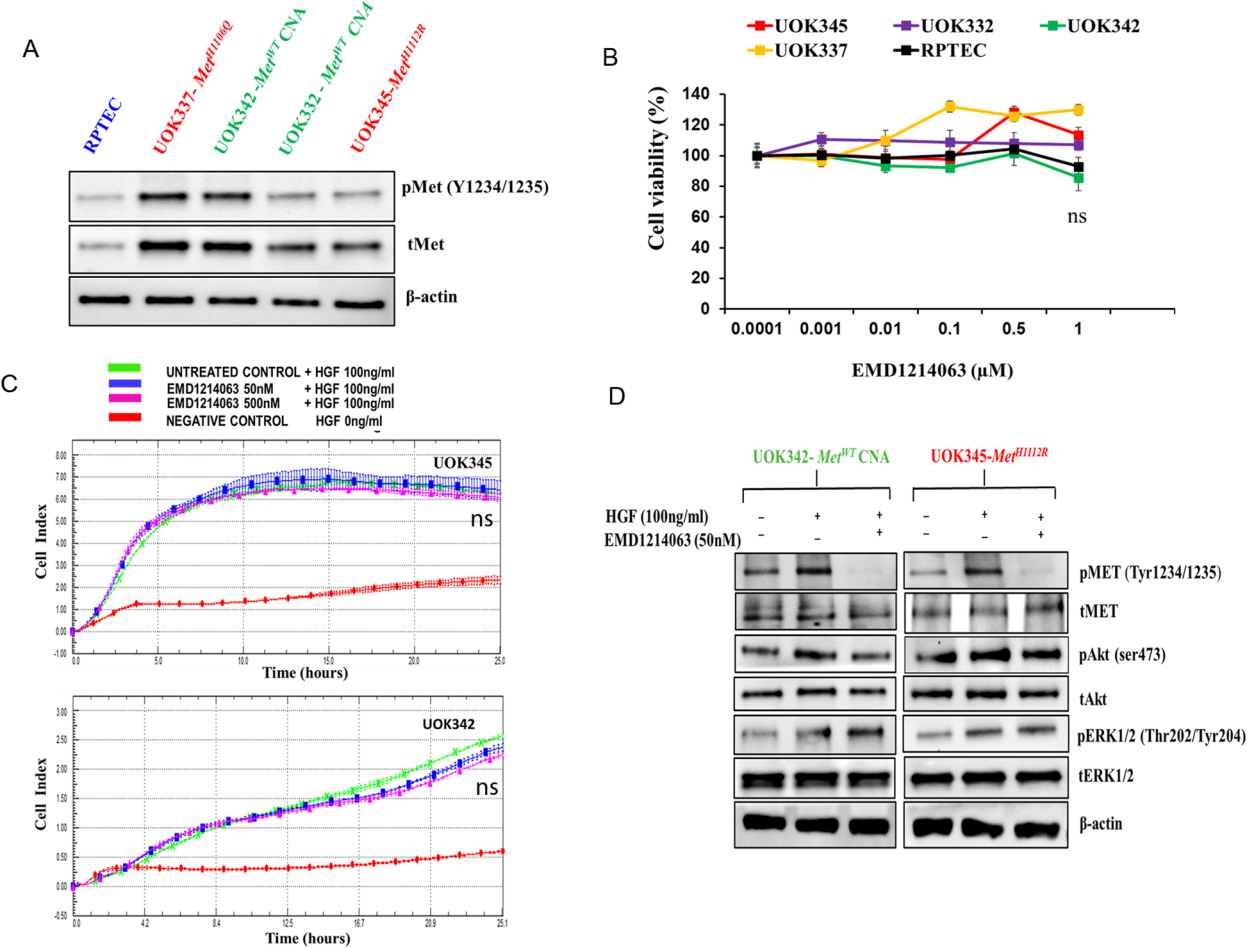
Increased levels of total and phosphorylated MET in PRCC cells. **A** Lysates from cell lines with gain of chromosome 7, harboring *MET* TK domain mutation (UOK345, H1112R; UOK337, H1106Q) or wild type *MET* (UOK342, UOK332) and normal RPTEC were collected and subjected to western blot analysis. Increased levels of both total and phosphorylated MET (Y1234/1235) were observed in all PRCC cell lines in comparison to RPTEC (**B**) Effect of selective MET TK inhibitor, EMD1214063 on viability of PRCC cells. UOK345, UOK337, UOK342, UOK332 and normal RPTEC cells were treated with a range of concentrations of EMD1214063 for 72 h in presence of 100 ng/ml HGF, and cell viability was determined using Cell-Titer Glo assay. EMD1214063 treatment had minimal effect on the proliferation of PRCC cell lines comparable to that seen in RPTEC cells. Data are expressed as mean ± SD of triplicates and are representative of three independent experiments. ns: not significant (p > 0.05), and was calculated by the two tailed Student’s t test (**C**) Effect of EMD1214063 on invasion in PRCC cells. Serum starved UOK345 or UOK342 cells were seeded in the upper chamber of CIM plate-16 in DMEM media containing 50 or 500 nM of EMD1214063 or vehicle. In the bottom chamber, DMEM with 100 ng/ml HGF or without HGF (negative control) were added and the inhibition of invasion relative to vehicle alone–treated cells was determined. The impedance value of each well was continually monitored every 15 min by the xCELLigence system for 24 h recorded as cell index. EMD1214063 treatment at either dose had no effect on the invasion of PRCC cell lines.. ns: not significant (p > 0.05), and was calculated by the two tailed Student’s t test. **D** Effect of EMD1214063 on phosphorylation of MET and downstream signaling molecules. EMD1214063 inhibits pMET(Y1234/1235) but no significant effect was observed on downstream signaling molecules pAKT (Ser473) and pERK (Thr202/Tyr204) in PRCC cell lines. UOK345 and UOK342 cells were treated with EMD1214063 (50 nM) for 2 h followed by the addition of HGF (100 ng/ml) for 15 min, lysed and subjected to western blot analysis. Three independent experiments were performed, and a representative result is shown. *p < 0.05 is considered significant and was calculated by the two tailed Student’s t test or other tests

UOK345, UOK337, UOK342, andUOK332 were generated in the Urologic Oncology Branch (UOB) from tumors derived from patients with metastatic PRCC, as described previously [21]. Briefly, extensive genomic characterization of these cell lines using DNA sequencing, spectral karyotyping and fluorescence in situ hybridization revealed the presence of either wild type (UOK342, UOK332) or activating mutations in the TK domain of *MET* (UOK345, H1112R; UOK337, H1106Q) [21]. Additionally, gain of chromosome 7 was demonstrated in all four cell lines [21] (Suppl. Table. S2). We confirmed increased levels of both total and phosphorylated MET (Y1234/1235) in all four PRCC cell lines compared to normal Renal Proximal Tubular Epithelial cells (RPTEC) using western blot analysis (Fig. 1A, Suppl. Fig. S1).

### MET tyrosine kinase inhibitor had minimal effects on growth and invasion of PRCC cells

Since MET has been implicated as a driver in at least a subset of PRCC tumors and is considered a clinically relevant target in these tumors, we evaluated the activity of the MET TKI EMD1214063 (Tepotinib) on PRCC cell lines in vitro. EMD1214063 is a potent and selective inhibitor of the MET TK receptor with > 1000-fold selectivity for MET compared to other kinases and is in phase 2 clinical trial for patients with confirmed *MET* exon 14 skipping mutation with advanced or metastatic non–small-cell lung cancer [28, 29]. We found that EMD1214063 had minimal effects on the proliferation of UOK345, UOK337, UOK342 and UOK332 cells, with growth inhibition comparable to that seen in RPTEC cells (Fig. 1B). Similar effects were observed when these cell lines were treated with other MET selective tyrosine kinase inhibitors (capmatinib and AMG208) (data not shown). Furthermore, PRCC cells treated with EMD1214063 in the presence of HGF (100 ng/ml) demonstrated no change in invasion when compared to vehicle alone-treated cells (Fig. 1C).

### Effect of EMD1214063 on MET kinase and downstream signaling pathways

Since auto-phosphorylation of MET on tyrosine residues Y1234/1235 in its C-terminal domain provides a docking site for recruitment of signal transducers that are necessary for activation of downstream signaling pathways including PI3K/AKT and MAPK/ERK [30], we examined the effect of EMD1214063 on phosphorylation of MET (Y1234/1235) and known downstream mediators of MET activity including AKT and ERK1/2 in PRCC cells. EMD1214063 (50 nM) markedly inhibited the auto-phosphorylation of MET as determined by western blot analysis (Fig. 1D). However, there was minimal effect on growth or invasion of these cells at this concentration and up to tenfold higher concentrations of EMD1214063 (Fig. 1B and C). Furthermore, there was no significant modulation of downstream mediators of MET activity, including phospho-AKT (Ser473) and phospho-ERK (Thr202/Tyr204) (Fig. 1D). Together, these results suggest that EMD1214063 has little effect on the growth of PRCC cell lines in vitro, probably attributable to the activation of the MAPK/ERK and PI3K/AKT pathways by alternative mechanisms.

### High Throughput drug screening identifies alternative therapeutic targets in PRCC cells

**Fig. 2:**
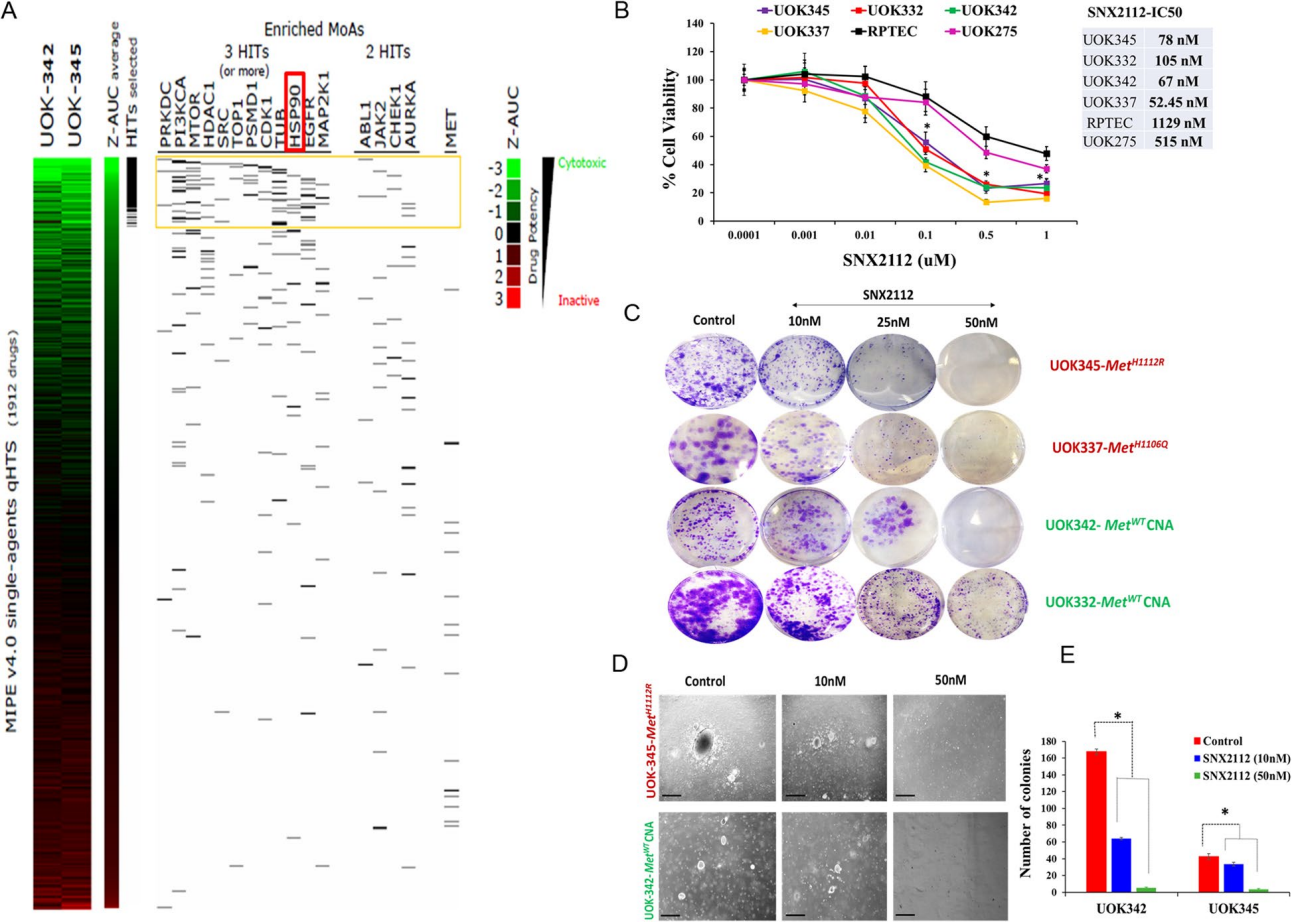
HSP90 inhibitor, SNX2112 induces tumor-suppressive effects in PRCC cells. **A** Heat map view of the high-throughput single-agent drug screening in PRCC cell lines (UOK345, UOK342). Z-transformed, area-under-the-curve (Z-AUC) values were used to assess compound potency and generate this heat-map. Heatmap ranking is based on the average compound potency in the two PRCC cell-lines tested. Highly potent HITs were defined as drugs whose Z-AUC value was < -1.5 in both cell lines (*n* = 110). Selected target-classes that were overrepresented among these HITs are highlighted on the top part of the plot. For any given target class, including HSP90 inhibitors, individual drugs are identified as individual black line and displayed accordingly to the average potency ranking. **B** Effect of SNX2112 on cell viability in PRCC. UOK345, UOK337, UOK342, UOK332 and normal RPTEC cell lines were seeded in 96 well plate in media containing 10% fetal bovine serum and incubated with a range of concentrations of SNX2112 for 72 h, and cell viability was determined using Cell-Titer Glo assay. Data are expressed as mean ± SD of triplicates and are representative of three independent experiments. *p < 0.05 is considered significant and was calculated by the two tailed Student’s t test. **C** SNX2112 dramatically inhibits 2D colony formation in PRCC cells. Representative images of colonies stained with crystal violet (0.5%) after 10–12 days treatment. **D** SNX2112 decreases 3D soft-agar colony formation in PRCC cells under anchorage-independent conditions. Representative images of colonies are shown on day 21 of treatment for each condition. Scale bars: 100 μm. **E** Bar graphs represent the number of soft agar colonies. Data represent mean ± SD of triplicate experiments *p < 0.05 is considered significant and was calculated by the two tailed Student’s t test or other tests

In order to uncover alternative therapeutic strategies for patients with papillary renal cell carcinoma, we tested the dose–response behaviors in two PRCC cell lines- one with a *MET* TK domain mutation (UOK345, H1112R) and a second with copy number gain of wild type *MET* (UOK342)- employing a MIPE library which was comprised of a panel of 1912 mechanistically annotated anti-cancer agents that are FDA approved or are currently being evaluated in clinical trials. As demonstrated in our growth inhibition assays (Fig. 1B), most MET inhibitors were characterized by weak antitumor activity in the high throughput screen (Fig. 2A). However, we identified several other classes of agents that displayed notable anti-tumor activity against both papillary cell lines (Fig. 2A). The ranking of these compounds was based on average potency obtained using Z-transformed, area-under-the-curve (Z-AUC) values for each compound (Fig. 2A). PI3KCA, mTOR, TUB, EGFR, HSP90, HDAC and PSMD1 inhibitors were among the most active group of agents in the HTS. Amongst these classes, we selected HSP90 inhibition as the target of this study with SNX2112 chosen as the representative HSP90 inhibitor, due to it having the greatest effect in the screen (Suppl. Fig. S2). Several factors informed our selection of HSP90 inhibition in addition to their effectiveness in the screening data. Firstly, HSP90 is involved in the folding and activation of specific client proteins including several oncologically relevant molecules downstream from MET, PI3K, mTOR and EGFR pathways, thus HSP90 inhibition may simultaneously target many of the pathways identified in the screen [17–19]. This is likely to provide a more effective response than specific, individual targeting of each of the other identified pathways. Secondly, addition of HSP90 inhibitors have been shown to overcome resistance to MET TKIs in MET driven tumor models and SNX2112 has been shown to be effective as a single agent in a cell line with acquired MET TKI resistance [19, 20]. SNX2112, via the prodrug SNX5422, is orally bioavailable and acts by competitively binding to the N-terminal adenosine triphosphate (ATP) binding site of HSP90, making it a useful reagent for in vivo studies [25].

### HSP90 inhibition results in potent growth inhibition of PRCC cells

We assessed the biologic effects of HSP90 inhibition in a panel of PRCC cell lines including those with *MET* mutations (UOK345, UOK337) and those with copy number gain of wild type *MET* (UOK342, UOK332). Treatment with SNX2112 exhibited potent and dose dependent growth inhibition in all PRCC cell lines evaluated, in vitro (IC50 range- 52.5 nmol/l to 105 nmol/l) compared to vehicle treated control (*p* < 0.05); however, 50% inhibition was not achieved even at higher, micromolar concentrations of the agent in normal renal tubular epithelium derived RPTEC cells (IC50-1129 nM). The PRCC cell line UOK275, was used as an additional wild type *MET* control [22] and was significantly less sensitive to SNX2112 treatment, with an IC50 (515 nM) that was 5 to tenfold higher than that seen with *MET* altered PRCC lines (Fig. 2B). Further, 2D colony foci formation was significantly suppressed (*p* < 0.05) when PRCC cells were seeded at low density (1 × 10^3^ – 1.5 × 10^3^ cells/well) and treated with SNX2112. (Fig. 2C). Similarly, 3D soft-agar colony formation under anchorage-independent conditions was potently inhibited with SNX2112 treatment at nanomolar concentrations (*p* < 0.05) (Fig. 2D, E).

### SNX2112 induces the degradation of HSP90 client proteins

**Fig. 3:**
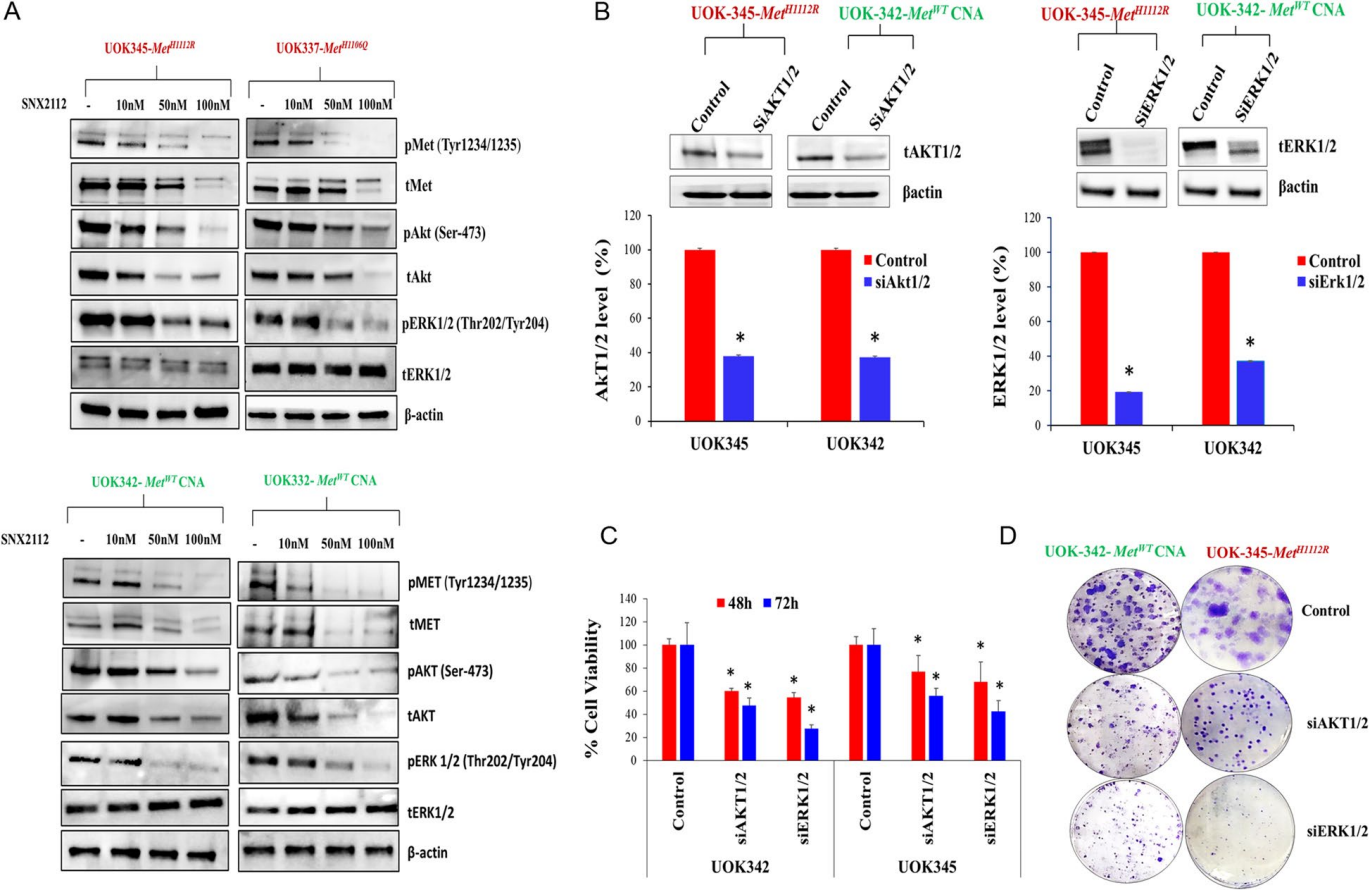
SNX2112 treatment leads to degradation of HSP90 client proteins in PRCC cells (**A**) PRCC cell lines (UOK345, UOK337, UOK342, UOK332) were treated with different concentrations of SNX2112 (10 nM, 50 nM and 100 nM) as indicated for 24 h, followed by Western Blot analysis. SNX2112 treatment induced the degradation of client proteins such as MET, AKT and inhibits phosphorylated forms of MET (Y1234/1235), AKT (Ser473) and ERK1/2 (Thr202/Tyr204). Three independent experiments were performed, and a representative blot is shown. **B** Knockdown of AKT1/2 and ERK1/2 proteins confirmed by western blot. PRCC cells with *MET* activating mutation (UOK345) or copy number gain of WT *MET* (UOK342) were transfected with siAKT1/2, siERK1/2 or non-targeting control, lysed and subjected to western blot. The percentage knockdown for AKT1/2 or ERK1/2 levels was calculated for cells treated with targeting siRNA for AKT1/2 or ERK1/2 relative to the cells treated with non-targeting control after being normalized with an internal control (β actin). Data are representative of three independent experiments. Bar graphs represent the mean ± SD, *n* = 3. *p < 0.05 is considered significant and was calculated by the two tailed Student’s t test. **C** AKT1/2 and ERK1/2 silencing decreases PRCC cell viability. UOK345 and UOK342 cells were transfected with siAKT1/2, siERK1/2 or non-targeting control and effect on cell viability was assessed at 48 h and 72 h post-transfection by Cell-Titer Glo assay. Data are mean ± SD of three independent experiments. *p < 0.05 is considered significant and was calculated by the two tailed Student’s t test. **D** AKT1/2 and ERK1/2 knockdown leads to diminished 2D-colony formation. Following transfection (48 h) with siAKT1/2, siERK1/2 and non-targeting control siRNA, UOK345 and UOK342 cells were trypsinized and seeded at very low density (1000–1500 cells/well) in six-well plates. Tumor foci were stained with crystal violet (0.5%) after 10–12 days and imaged with microscopy. *p < 0.05 is considered significant and was calculated by the two tailed Student’s t test or other tests

Since HSP90 stabilizes numerous oncogenic client proteins [31], we sought to assess the effects of SNX2112 on the expression of relevant proteins such as MET, pMET (Y1234/1235) and downstream mediators of MET activity, including phosphorylated forms of AKT and ERK1/2 in PRCC cells. We observed that SNX2112 treatment led to degradation of total MET and inhibited pMET (Y1234/1235) in a dose-dependent manner (Fig. 3A). However, unlike TKIs targeting MET, SNX2112 also induced the degradation of other HSP90 client including the native form of AKT while also inhibiting phosphorylated forms of AKT (Ser473) and ERK (Thr202/Tyr 204) (Fig. 3A). In order to better understand if SNX2112 induces degradation and/or decreases protein stability of these proteins, cells pre-treated with the protein synthesis inhibitor cycloheximide (10ug/ml) alone or in combination with the proteasome inhibitor MG132 (10uM) were then exposed to SNX2112 (100 nM) or vehicle for an additional 8 h. In the absence of MG132, SNX2112 resulted in markedly reduced half-life compared to untreated cells of the HSP90 client proteins tMET, pMET, tAKT, pAKT, and pERK1/2. However, in the presence of MG132, we observed protection of these HSP90 clients from the destabilizing effect of SNX2112, suggesting that SNX2112 induces proteasome-dependent degradation of these proteins (Suppl. Fig. S3). These data indicate that the potent activity of SNX2112 against PRCC may be the result of more complete abrogation of both MET and key downstream mediators of MET activity that are not adequately inhibited by MET TKIs.

### Biologic effects of SNX2112 are partially dependent on PI3K/AKT and MEK/ERK1/2 signaling

To elucidate whether the anti-tumor effects of SNX2112 in PRCC cells are dependent on the PI3K/AKT and MEK/ERK1/2 signaling axis, cells with *MET* mutation (UOK345) as well as those with copy number gain of wild type *MET* (UOK342) were transfected with small interfering RNA (siRNA) for AKT1/2, ERK1/2 or non-targeting control. Reduced expression of AKT1/2 and ERK1/2 proteins was confirmed in the siAKT1/2 and siERK1/2 transfected cells respectively compared to non-targeting control by western blot (Fig. 3B). The percentage decrease in expression for AKT1/2 and ERK1/2 proteins was between 60–80% in UOK345 and UOK342 cell lines as indicated in Fig. 3B. The reduction of either endogenous AKT1/2 or ERK1/2 led to a significant decrease in PRCC cell viability at 48 h and 72 h (*p* < 0.05) (Fig. 3C). A marked decrease in 2-D colony formation was also seen in both PRCC cell lines when expression of AKT1/2 or ERK1/2 were knocked down (Fig. 3D). To further determine the role played by these pathways in SNX2112-mediated growth inhibition, we evaluated the efficacy of SNX2112 in PRCC cells (UOK345 and UOK342) overexpressing AKT1/2 or ERK1/2. PRCC cell lines overexpressing AKT1/2 or ERK1/2 were marginally less sensitive to SNX2112 treatment than parent cells (not transfected with AKT1/2 or ERK1/2 expression plasmids) (Suppl. Fig. S4). These data suggest that AKT1/2 or ERK1/2 overexpression only partially abrogates SNX2112 biologic effects. These results are concordant with our HTS data which identified up- and downstream regulators of AKT1/2 (PI3KCA and mTOR) and the up-stream components of the ERK1/2 pathway (SRC, EGFR, MAP2K1) as targets of interest. These data further suggest that, while PI3K/AKT and MEK/ERK1/2 signaling are crucial for growth of these PRCC cells, the anti-tumor activity of SNX2112 likely involves important additional signaling pathways.

### SNX2112 treatment induces apoptosis and G2/M cell cycle arrest in PRCC cells

HSP90 client proteins such as PI3K/AKT and MEK/ERK1/2 are known to regulate cell death and apoptotic pathways as well progression through the cell cycle [32]. We sought to determine the extent to which growth inhibition of PRCC cells was mediated by apoptosis and/or cell cycle arrest. PRCC cell lines were treated with SNX2112 for 48 h and changes in the proportion of apoptotic cells and markers of apoptosis (caspases 3/7 and PARP) were analyzed.

**Fig. 4:**
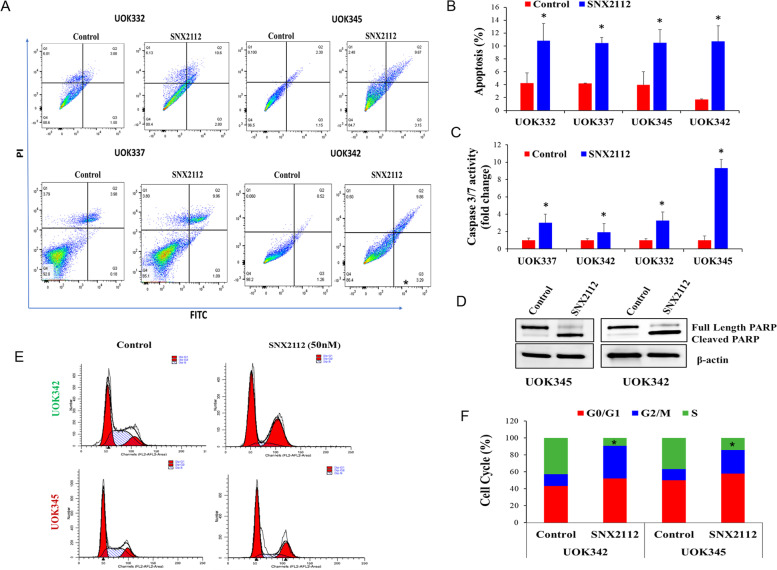
SNX2112 treatment induces apoptosis and causes G2/M cell cycle arrest in PRCC cells. UOK345, UOK337, UOK342 and UOK332 cell lines were treated with SNX2112 (50 nM) or vehicle for 48 h, stained with annexin V and propidium iodide and analyzed using flow cytometry (**A**) Representative dot blots and (**B**) bar graphs expressing increased percentage of apoptotic cells after SNX2112 (50 nM) treatment. Data are mean ± SD of three independent experiments. *p < 0.05 is considered significant and was calculated by the two tailed Student’s t test. **C** SNX2112 treatment is associated with increased caspase 3/7 activity. UOK345, UOK337, UOK342 and UOK332 cell lines were treated with SNX2112 (50 nM) or vehicle control for 48 h and caspase 3 and 7 activity was detected by luminescent assay. Data are mean ± SD of triplicate experiments *p < 0.05 is considered significant and was calculated by the two tailed Student’s t test. **D** SNX2112 treatment increases levels of cleaved PARP. PRCC cell lines (UOK345, UOK342) treated with 50 nM of SNX2112 for 48 h were lysed and subjected to western blot analysis. Three independent experiments were performed, and a representative blot is shown. **E** SNX2112 treatment causes G2/M cell cycle arrest. UOK345 and UOK342 cells were treated with 50 nM of SNX2112 for 48 h, stained with propidium iodide, followed by flow cytometric analysis of the cell cycle distribution. **F** Histogram representation of the percentage of cells in different cell-cycle phases. *p < 0.05 is considered significant and was calculated by the two tailed Student’s t test

SNX 2112 treatment resulted in a significant (*p* < 0.05) increase in the number of cells (expressed in %) undergoing apoptosis [control vs treated: 3.9 vs 10.48% (UOK345); 1.7 vs 10.73% (UOK342); 4.19 vs 10.43% (UOK337); 4.2 vs 10.8% (UOK332) (Fig. 4A and B). SNX2112 treatment also significantly (*p* < 0.05) increased the activity of caspases 3 and 7 at 48 h (Fig. 4C). Since cleavage and inactivation of PARP by caspases is a hallmark of apoptosis [33], we assessed the effect of SNX2112 on PARP cleavage following 48 h of treatment. Western blot analysis revealed increased levels of cleaved PARP, which further confirmed the induction of apoptosis in SNX2112 treated cells (Fig. 4D).

To evaluate the effects of SNX112 on the cell cycle, cells with *MET* mutation (UOK345) as well as those with copy number gain of wild type *MET* (UOK342) were treated with 50 nM of SNX2112, stained with propidium iodide and analyzed by flow cytometry. As shown in Fig. 4E and F, SNX2112 led to a significant (*p* < 0.01) G2/M arrest in both PRCC cell lines. Collectively, these results suggest that SNX2112 treatment induced apoptosis as well as G2/M arrest in PRCC cells.

### Identification of differentially expressed genes by RNA sequencing and additional pathways affected by SNX2112 using gene set enrichment analysis (GSEA)

**Fig. 5:**
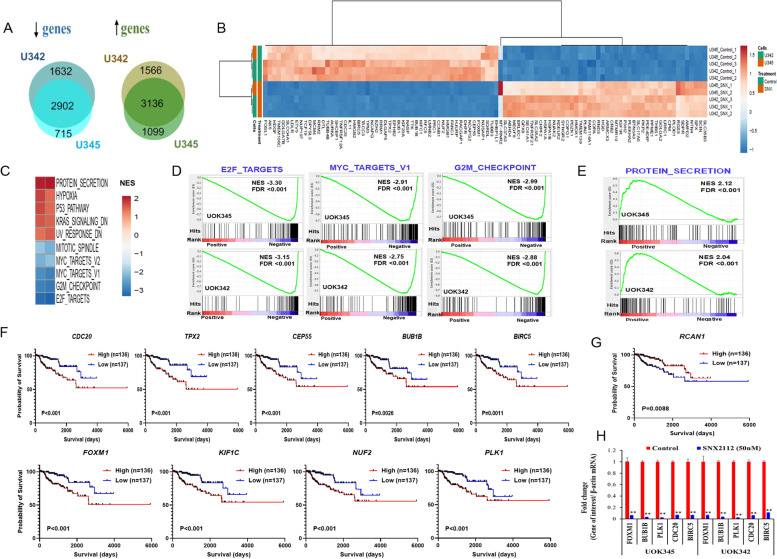
RNA sequencing of PRCC cells treated with SNX2112 (**A**) Venn diagram showing the number of differentially expressed genes in UOK345 and UOK342 following treatment with SNX2112. UOK345 and UOK342 cells were treated with SNX2112 (50 nM) and RNA was isolated after 48 h of treatment for the mRNA-Seq analysis. Of the 6,038 differentially expressed genes common in both cell lines, 2,902 genes were downregulated (fluorescent blue color), and 3,136 genes were upregulated (dark green color). Analysis used genes meeting an FDR value < 0.05; fold change > 1.5 threshold. **B** Heatmap showing the mRNA levels of the top 100 genes (50 up or downregulated) after SNX2112 (50 nM) treatment. The expression levels of each gene were normalized to the total mRNA abundance of each sample and compared with that of vehicle-treated controls. **C** The top ranked negatively, and positively enriched gene sets identified using Gene Set Enrichment Analysis (GSEA) in response to SNX2112. GSEA was performed with top common differentially expressed genes in UOK345 and UOK342 cells after SNX2112 treatment using 50 HALLMARK gene sets database in MSigDB. GSEA plots showing strong (**D**) negative enrichment of the E2F, G2M_checkpoint and MYC Targets v1 and (**E**) positive enrichment of protein secretion pathway in UOK345 and UOK342 cells in response to SNX2112. NES; Normalised Enrichment Score, FDR: false discovery rate (**F**) Kaplan–Meier estimate of overall survival based on expression of differentially expressed genes (TCGA PRCC Cohort). Relative high expression of *CDC20, TPX2, CEP55, FOXM1, KIFC1, NUF2, BUB1B, BIRC5* and *PLK1* genes and (**G**) low expression of *RCAN1* gene was associated with poor overall survival in KIRP cohort. Log rank (Mantel-Cox) test was used for significance; *p* < 0.05 was considered significant. **H** Relative mRNA expression of selected E2F, G2M checkpoint and MYC regulated genes. The mRNA levels of *CDC20, FOXM1, BUB1B, BIRC5* and *PLK1* genes were evaluated by qRT-PCR 48 h after SNX2112 treatment (50 nM) in UOK345 and UOK342. Data are mean ± SEM of three independent experiments. **p < 0.001, Statistical significance was determined using the two tailed Student’s t test

To study the effects of SNX2112 on global gene expression in PRCC, cells with *MET* mutation (UOK345) and/or copy number gain of wild type *MET* (UOK342) were treated with SNX2112 (50 nM) for 48 h and mRNA-sequencing was performed to identify differentially expressed genes (DEGs). The expression of approximately 6,038 genes was significantly altered (false discovery rate [FDR] q < 0.05; fold change > 1.5) in both cell lines treated with SNX2112 compared to vehicle controls (Fig. 5A, Suppl. Table. S3). Of the 6,038 differentially expressed genes, 2,902 genes were downregulated while 3,136 genes were upregulated (Fig. 5A). The heatmap (Fig. 5B) represents the top 100 genes (50 up- and down) modulated by SNX2112 treatment in both cell lines. These genes were ranked by their false discovery rate and fold change between SNX2112 treated versus vehicle treated cells.

Next, GSEA were performed using the 50 Hallmark gene set collections in MSigDB for the identification of specifically enriched biological pathways following SNX2112 treatment. GSEA analysis of the common differentially expressed genes in both *MET* mutated and WT *MET* cell lines treated with SNX2112 revealed strong negative enrichment for the gene sets involved in cell cycle progression and proliferation including hallmark_E2F_targets (FDR =  < 0.001; NES ≥ 3), hallmark_G2M_checkpoint (FDR =  < 0.001; NES ≥ 2) and hallmark_MYC_targets_V1 (FDR =  < 0.001; NES ≥ 2) (Fig. 5C and D). Strong positive enrichment of gene sets involved in hallmark_protein_secretion (FDR =  < 0.001; NES ≥ 2) was also observed (Fig. 5E). The upregulated and downregulated gene sets are presented in Fig. 5C and tabulated with FDR q value in Suppl. Table. S4.

### SNX2112 treatment downregulates unique PRCC-associated prognostic genes

In order to determine the biologic relevance of the genes in PRCC that were modulated by SNX2112, likely due to the downstream consequences of degradation of HSP90 clients, we explored the TCGA dataset from the Kidney Renal Papillary Cell Carcinoma (KIRP) cohort to ascertain whether any of the top 200 (100 up and down) differentially expressed genes identified by RNA seq analysis (Suppl. Table. S3) were aberrantly expressed and/or associated with outcome. Several genes downregulated following SNX112 treatment, including *CDC20, TPX2, CEP55, FOXM1, KIFC1, NUF2, BUB1B, BIRC5* and *PLK1* were overexpressed in Papillary Renal Cell Carcinoma TCGA cohort (Suppl. Fig. S5). Consistent with observations from the TCGA dataset RNA seq gene expression demonstrated overexpression of these genes in the PRCC cell line with relatively low expression observed in RPTEC cells (Suppl. Table. S5). Kaplan–Meier survival analysis were performed for each of these genes according to its relative expression level, dichotomized as high or low relative to its median expression among the entire TCGA KIRP cohort. Interestingly, high expression of each of these genes was associated with poor overall survival (*p* < 0.001) (Fig. 5F). Additionally, *RCAN1*, which figured in the list of the top 100 upregulated genes, demonstrated decreased expression in PRCC (Suppl. Fig. S5), with lower expression predicting poor overall survival in the KIRP TCGA cohort (*p* < 0.01) (Fig. 5G). Notably, *RCAN1* has been reported as a tumor suppressor in many cancers, including thyroid and breast cancers [34, 35].

Of the genes that were downregulated in response to SNX2112 and also appear to be overexpressed and associated with poor prognosis in PRCC, five genes (*CDC20, FOXM1, BUB1B, BIRC5* and *PLK1*) that were components of the most enriched pathways identified by GSEA (E2F, G2/M checkpoint and MYC) were selected for further study. All five genes have previously been shown to be involved in progression of a variety of cancers including prostate, bladder, and gastric cancer [36–41]. mRNA levels of all five genes in UOK345 or UOK342 cell lines treated with SNX2112, and vehicle treated controls were analyzed using quantitative Real Time PCR. Expression of all five genes was significantly downregulated in PRCC cell lines treated with SNX2112 compared to vehicle controls (Fig. 5H), a finding consistent with that seen in RNA-seq analysis.

### HSP90 inhibition suppresses growth of PRCC xenografts *in vivo*

**Fig. 6:**
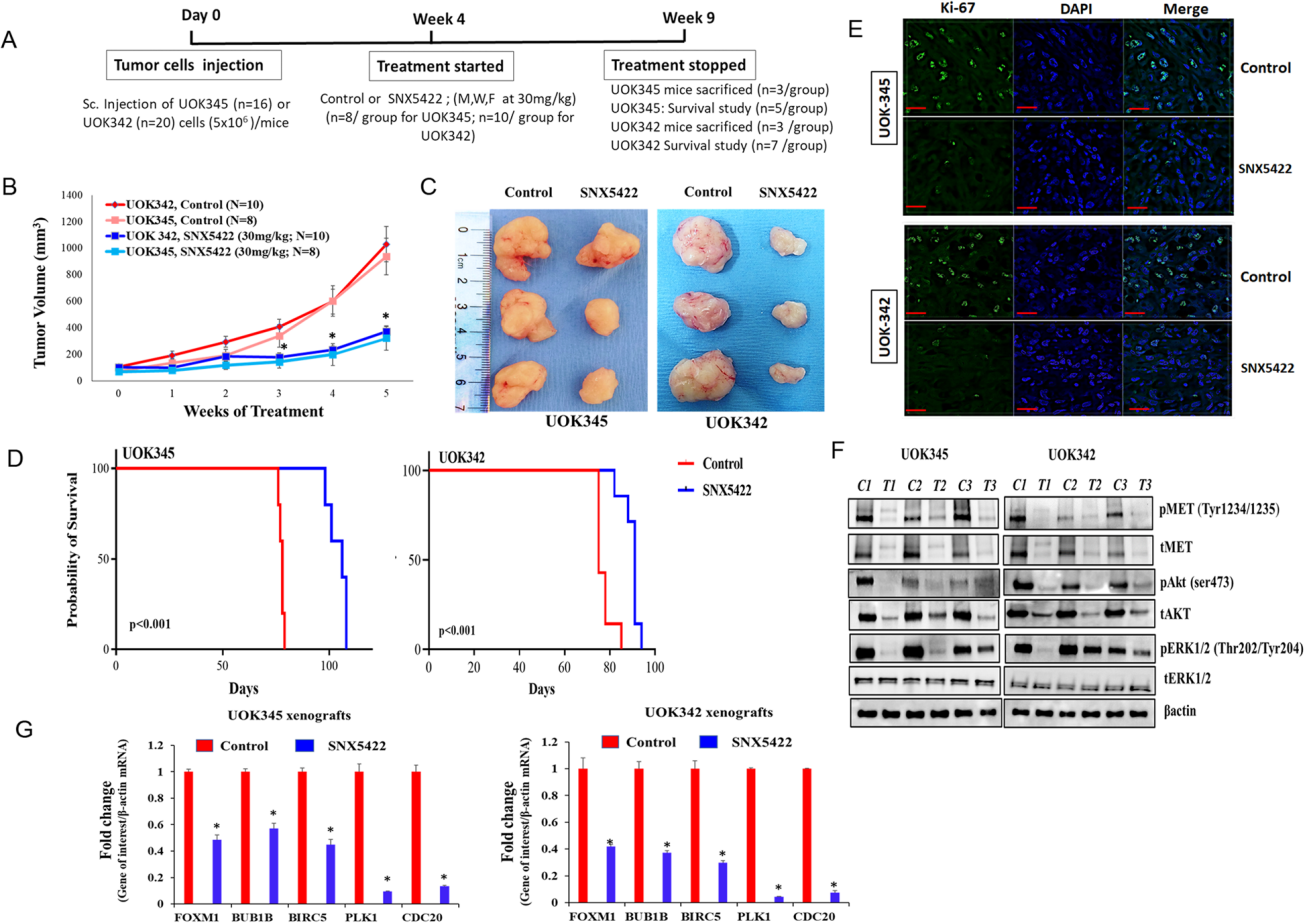
SNX5422 suppresses tumor growth in PRCC xenografts (**A**) The flanks of NSG mice were implanted with UOK345 or UOK342 cells to establish subcutaneous xenografts. Approximately four weeks following injection, mice bearing tumors were randomized and divided into 2 groups (UOK345: *n* = 8, each group; UOK342: *n* = 10, each group) and treated with either SNX5422 (30 mg/kg week, 3 times a week, for 5 weeks) or vehicle control. **B** The graph depicts changes in tumor volume (mm3) over 5 weeks of treatment. Data represented as mean ± SEM. **C** Photograph showing excised tumor xenografts in control and treated mice (*n* = 3, each group) after 5 weeks of treatment. **D** Kaplan–Meier survival curve of mice treated with SNX5422 or control vehicle in which a log rank (Mantel-Cox) test was used for significance; *p* < 0.05 was considered significant. The experimental end point (maximum tumor volume) was considered for KM survival plot. **E** Ki-67 (proliferation) levels were assessed by immunohistofluorescence in tumors of SNX5422 or control-treated mice. Scale bars: 20 μm. **F** Expression of MET and AKT in UOK345 and UOK342 tumor xenogratfs. Excised tumor xenografts of SNX5422 treated (T1, T2 and T3; *n* = 3) and control (C1, C2, C3; *n* = 3) mice were lysed and subjected to western blotting (**G**) Relative mRNA expression of *CDC20, FOXM1, BUB1B, BIRC5* and *PLK1* genes in UOK345 and UOK342 tumor xenogratfs. RNA was isolated from excised tumor xenografts of SNX5422 treated and control mice for qRealTime-PCR. Data are presented as mean ± SEM (*n* = 3 mice, each group); *p < 0.05 is considered significant and was calculated by two tailed Student’s t test or other tests

To evaluate the in vivo anti-tumor efficacy of HSP90 inhibitors in a mouse model using PRCC xenografts, NSG mice were injected subcutaneously either with UOK345 (*MET* mutation) or with UOK342 (copy number gain of wild type *MET*) and treated with SNX5422 (30 mg/kg) 3 times a week until five weeks once tumors were established (100 mm^3^) as depicted in Fig. 6A. SNX2112 is the active inhibitor but SNX 5422 is a prodrug that is orally available and used in animal studies [19, 25, 26].

SNX5422 treatment significantly inhibited the growth of UOK345 and UOK342 xenografts over the five weeks of treatment compared to the vehicle treated control group (*p* < 0.01) (Fig. 6B and C). Furthermore, mice treated with SNX5422 demonstrated a marked improvement in overall survival (median OS 91 days for UOK342 and median OS 106 days for UOK345) compared to the vehicle treated control mice (median OS 76.5 days for UOK342 and median OS 78 days for UOK345) (*p* < 0.001) (Fig. 6D).

Next, the xenograft tumors were examined by immunohistofluorescence to detect the levels of Ki-67, a proliferation marker. As shown in Fig. 6E, SNX5422 treatment resulted in significant decrease in Ki-67 levels. Further, SNX5422 also induced the degradation of MET and inhibits key downstream mediators of MET activity including activated forms of MET(Y1234/1235), AKT (Ser473) and ERK (Thr202/Tyr 204) in tumor-bearing xenografts (*n* = 3, each group) as demonstrated by western blotting (Fig. 6F). Interestingly, the expression of all five genes associated with PRCC survival including *CDC20, FOXM1, BUB1B, BIRC5* and *PLK1* was significantly reduced in SNX5422 treated (*n* = 3) compared to control mice (*n* = 3) (*p* < 0.001) (Fig. 6G).

## Discussion

Papillary renal cell carcinoma is the second most common type of RCC, and accounts for 15–20% of all kidney cancer diagnoses [1]. While a number of highly effective treatment strategies are available for treating patients with clear cell RCC, there are relatively few options for those with advanced PRCC and their prognosis remains poor [10–13]. Identification of alternative strategies that are effective in PRCC is an area of unmet medical need.

Pharmacologic interventions targeting MET have been the subject of clinical investigation in patients with advanced PRCC, based on the identification of germline or somatic *MET* alterations in a subset of patients [5–9]; additionally, the high prevalence of chromosome 7 gain in papillary tumors [5], has led to speculation that copy number gain of wild type *MET* (which is located on chromosome 7) might lead to MET-dependent oncogenesis even in the absence of activating *MET* mutations. Our data demonstrate that inhibition of MET tyrosine kinase activity is associated with modest activity in PRCC cell lines with *MET* mutations as well as those with copy number gain of WT *MET*; this is likely due, at least in part, to persistent activation of downstream signaling pathways such as MEK/ERK1/2 and PI3K/AKT. In previous studies, activation of redundant survival pathways or drug-resistant kinase mutations have been implicated in mediating resistance to targeted MET inhibition in *MET*-amplified tumors [19, 20]. Our experience in preclinical models is echoed in results from multiple clinical studies evaluating MET targeted tyrosine kinase inhibitors (including multitargeted tyrosine kinase inhibitors with activity against MET, such as cabozantinib), which have demonstrated only modest activity in patients with PRCC [10, 11, 13–16].

In an effort to identify new targets amenable to pharmacologic interdiction, we sought to explore alternative approaches. Using an unbiased screen, we identified HSP90 as a potential target of interest. MET and several downstream mediators of MET activity are known HSP90 clients [20, 31]. In addition, HSP90 inhibitors have been reported to overcome resistance against MET TKIs in MET-driven tumor models of gastric and lung cancer [19, 20]. In the present study we found that SNX2112, a representative HSP90 inhibitor, strongly inhibits growth of PRCC lines by inducing marked caspase-dependent apoptosis and G2/M cell-cycle arrest, effectively degrades MET, and downregulates downstream mediators of MET biological activity (including native and phosphorylated forms of AKT1/2 and ERK1/2), in PRCC-derived cell lines. SNX5422, an orally available prodrug of SNX2112, has been safely tested in multiple phase I studies in patients with solid tumors and hematological malignancies [42, 43]; SNX5422 is structurally and pharmacologically distinct from HSP90 inhibitors derived from the natural product geldanamycin, including17AAG [44] which was the first HSP90 inhibitor to enter the clinic. SNX5422 effectively inhibited the growth of PRCC xenografts in mice and prolonged survival of tumor bearing mice.

A host of cellular proteins mediating important functions are HSP90 clients and HSP90 inhibitors consequently modulate the expression of a diverse array of cellular proteins and signaling pathways [30, 45]. Using a series of knockdown experiments targeting selected pathways of interest, we demonstrated that the anti-tumor activity of SNX2112 was mediated at least in part by down-regulation of components of the PI3K/AKT and MEK/ERK1/2 pathways. We focused specifically on these pathways as SNX2112 antitumor activity has been shown to be mediated by PI3K/AKT and MEK/ERK1/2 pathways in other cancers including MET-amplified gastric and lung cancers, HER kinase dependent cancers, multiple myeloma and other hematologic tumors [19, 25, 26]; however, it is entirely possible that other HSP90 clients play additional roles in mediating the anti-tumor effects we observed. Additionally, RNA-seq-based global gene expression and gene set enrichment analyses identified several important new gene sets that are modulated by SNX2112 treatment in PRCC, including those regulated by E2F and MYC as well as genes regulating the G2M transition. A subset of five genes (*CDC20, FOXM1, BUB1B, BIRC5* and *PLK1),* whose overexpression is associated with poor prognosis in PRCC, was chosen for further validation and shown to be downregulated both in vitro in PRCC cell lines and in vivo in mice bearing PRCC xenografts following pharmacologic inhibition of HSP90. Of note, these genes have previously been reported to be involved in progression of a variety of cancers [36–41].

These data have helped uncover novel targets that merit further evaluation in PRCC. While the clinical utility of earlier HSP90 inhibitors like 17 AAG appears to be limited (the agent was evaluated in a small phase 2 study of PRCC and demonstrated no objective responses) [46, 47], newer, synthetic HSP90 inhibitors are now available and appear to be clinically active as well as offering a more acceptable safety profile. The HSP90alpha/beta-specific inhibitor, TAS-116 (pimitespib) has recently been reported to meet its Phase 3 endpoint by significantly prolonging progression-free survival in patients with gastrointestinal stromal tumor refractory to imatinib, sunitinib and regorafenib in a Phase-III clinical trial [48]; (Len Neckers, personal communication, 2021), and merits evaluation in PRCC. Additionally, our work suggests that the PI3K/AKT and MEK/ERK1/2 pathways may play an important role in at least some forms of papillary kidney cancer. Pharmacologic inhibitors of these pathways including PI3K/mTOR inhibitors and MEK inhibitors are already FDA-approved for several indications or are under clinical investigation and are currently undergoing further evaluation in preclinical models of PRCC in our laboratory. These data also suggest a role for E2F regulated genes in mediating the antitumor activity of HSP90 inhibitors. Furthermore, loss of *CDKN2A*, which would be predicted to result in CDK4/6 upregulation and dysregulation of E2F-dependent transcription and cell cycle checkpoints, is relatively common in papillary RCC [5, 49]. Consequently, agents that perturb E2F function, such as CDK4/6 inhibitors, are also under investigation in preclinical models. Indeed, combination of CDK4/CDK6 inhibition with the HSP90 inhibitor, Pimitespib has demonstrated additive anti-cancer effect in several solid tumor preclinical models, leading to the development of a Phase I trial of Palbociclib and Pimitespib in advanced breast cancer progressing on Palbociclib alone and other treatment-refractory solid tumors [50]. 

PRCC is refractory to most treatment options currently available to patients with other forms of kidney cancer (such as clear cell RCC) and the identification of effective therapeutic strategies would serve an important unmet medical need. While MET inhibition has been a major area of clinical investigation in patients with advanced PRCC, to date it has yielded only modest returns. Our work helps explain the limited clinical utility of MET inhibitors in these tumors and offers insights that have the potential for translation into clinically meaningful interventions.

## Supplementary Information

**Additional file 1:Supplementary Fig. S1.** Quantification of phospho-MET (pMET) and total-MET (tMET). **Supplementary Fig. S2.** Top 110 drugs selected based on potency in both PRCC cell lines by high-throughput single-agent drug screening. **Supplementary Fig. S3.** Effect of SNX2112 treatment on the stability/degradation of HSP90 client proteins. **Supplementary Fig. S4.** Evaluation of SNX2112 efficacy on PRCC cells overexpressing AKT1/2 or ERK1/2. **Supplementary Fig. S5.** Expression of genes in Papillary Renal Cell Carcinoma TCGA (KIRP) cohort. **Supplementary Table S1.** List of qRT-PCR TaqMan primer probes (assays) used for gene expression analysis. **Supplementary Table S2.** The genetic characterization of PRCC cell lines. **Supplementary Table S5.** Relative gene expression in normal (RPTEC) and PRCC cell lines (UOK345, UOK342) by RNA sequencing.

**Additional file 2:Supplementary Table S3.** Differentially expressed genes after SNX2112 treatment in UOK345 and UOK 342 cell lines by RNA seq.

**Additional file 3:Supplementary Table S4.** Gene set enrichment analysis (GSEA) analysis.

## Data Availability

Our RNA-seq data used in this study (RNA-seq before and after SNX2112 treatment) have been deposited in NCBI GEO database (GSE180820).

## Abbreviations

PRCC: Papillary renal cell carcinoma
HPRC: Hereditary papillary renal cell carcinoma
HSP90: Heat shock protein 90
PI3K: Phosphotidylinositol-3-Kinase
MET: Hepatocyte Growth Factor Receptor
HGF: Hepatocyte Growth Factor
RTK: Receptor Tyrosine Kinase
AKT: Protein kinase B
MEK: Mitogen-Activated Protein Kinase Kinase
MAPK1: Mitogen-Activated Protein Kinase Kinase 1
ERK1/2: Extracellular signal-regulated protein kinase 1/2
E2F: E2F Transcription Factor 1
MYC: MYC Proto-Oncogene
EGFR: Epidermal Growth Factor Receptor
VEGFR: Vascular Endothelial Growth Factor Receptor
CDK: Cyclin-Dependent Protein Kinases
ALK: Anaplastic Lymphoma Receptor Tyrosine Kinase
RAF: RAF Proto-Oncogene Serine/Threonine-Protein Kinase
PARP: Poly (ADP-ribose) polymerase
WT: Wild type
TKI: Tyrosine kinase inhibitor
HTS: High throughput screening
cDNA: Complementary Deoxyribonucleic Acid
RNA seq: RNA Sequencing
FDR: False discovery rate
DEG: Differentially expressed genes
MSigDB: Molecular Signatures Database
GSEA: Gene set enrichment analysis
qRT-PCR: Quantitative Real-time Polymerase Chain Reaction
ATCC: American Type Culture Collection
TCGA: The Cancer Genome Atlas
KIRP: Kidney Renal Papillary Cell Carcinoma
RPTEC: Renal proximal tubule epithelial cell
DMEM: Dulbecco's Modified Eagle Medium
FITC: Fluorescein isothiocyanate
Ki67: Marker of Proliferation Ki-67
DAPI 4′: 6-Diamidino-2-phenylindole
FDA: Food and Drug Administration
PFS: Progression Free Survival
ORR: Overall response rate
