# Supplementary material for: Inhibition of HSP 90 is associated with potent anti-tumor activity in Papillary Renal Cell Carcinoma
Source: J Exp Clin Cancer Res. 2022 Jun 27;41:208. doi: 10.1186/s13046-022-02416-z (PMC9235180; doi:10.1186/s13046-022-02416-z)
Supplement: Supplementary file 1 — Additional file 1:Supplementary Fig. S1. Quantification of phospho-MET (pMET) and total-MET (tMET). Supplementary Fig. S2. Top 110 drugs selected based on potency in both PRCC cell lines by high-throughput single-agent drug screening. Supplementary Fig. S3. Effect of SNX2112 treatment on the stability/degradation of HSP90 client proteins. Supplementary Fig. S4. Evaluation of SNX2112 efficacy on PRCC cells overexpressing AKT1/2 or ERK1/2. Supplementary Fig. S5. Expression of genes in Papillary Renal Cell Carcinoma TCGA (KIRP) cohort. Supplementary Table S1. List of qRT-PCR TaqMan primer probes (assays) used for gene expression analysis. Supplementary Table S2. The genetic characterization of PRCC cell lines. Supplementary Table S5. Relative gene expression in normal (RPTEC) and PRCC cell lines (UOK345, UOK342) by RNA sequencing. [file 13046_2022_2416_MOESM1_ESM.docx]

**Supplementary Fig. S1. Quantification of phospho-MET (pMET) and total-MET (tMET)**

**
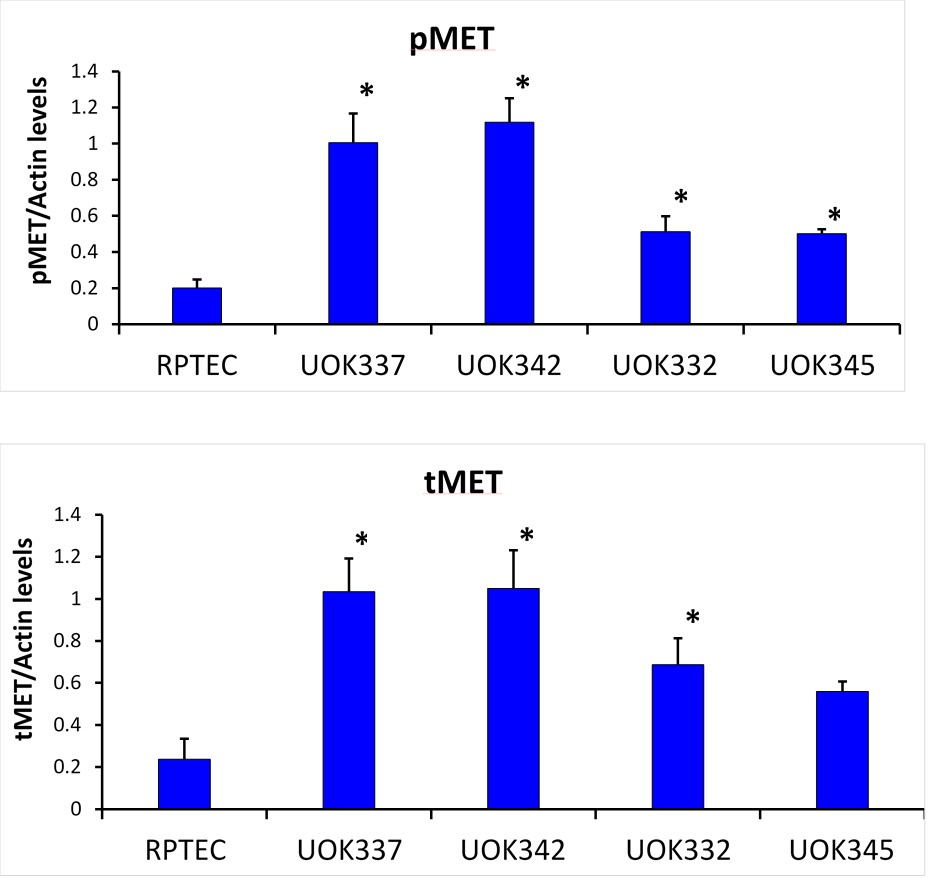
**

*****

The bar graph represents the relative protein abundance of pMET and tMET depicted in the western blot using densitometry analysis. Data are presented as mean ± SEM, n=3. *p < 0.05, p < 0.05 is considered significant and was calculated by the two-tailed Student’s t test.

**Supplementary Fig. S2. Top 110 drugs selected based on potency in both PRCC cell lines by high-throughput single-agent drug screening**

**
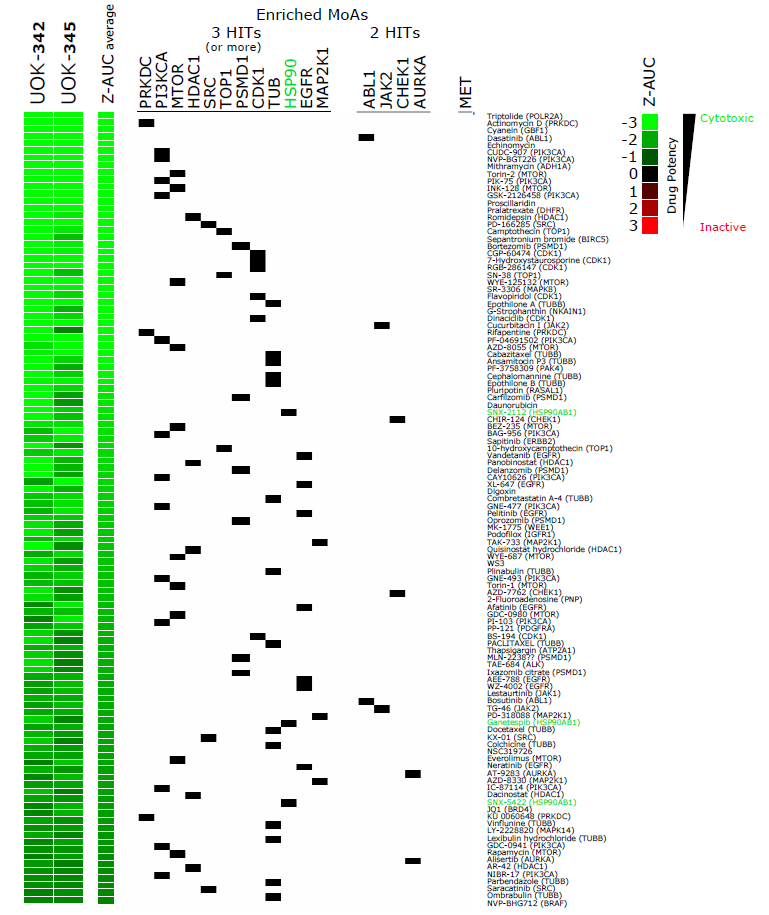
**

**Heat map showing results of top 110 drugs with notable anti-tumor activity against both papillary renal cell carcinoma cell lines (UOK345, UOK342)**. The ranking of these compounds was based on average potency obtained using Z-transformed, area-under -the-curve values (Z-AUC <-1.5) for each compound. The HSP90 inhibitors are highlighted in green.

**Supplementary Fig. S3. Effect of SNX2112 treatment on the stability/degradation of HSP90 client proteins**

**
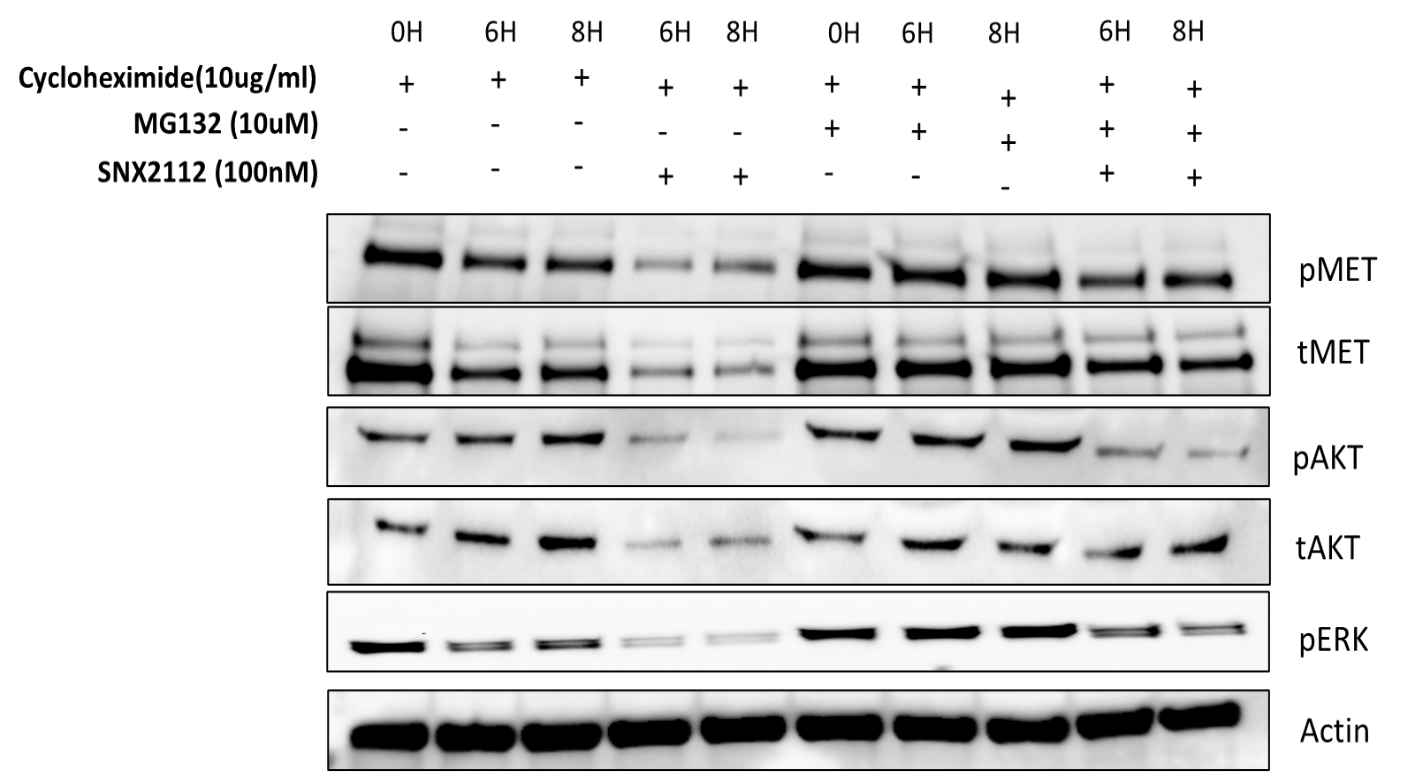
**

**Estimation of tMET, pMET, tAKT, pAKT and pERK1/2 protein levels by western blotting**. Protein levels were measured in UOK345 cell lysate, pre-incubated with 10 μM MG132 and/or 10 μg/mL cycloheximide (CHX) for 1 hour, followed by +/- 100 nM SNX2112 treatment up to 8 additional hours. Equal amounts of protein (20ug) were loaded.

**Supplementary Fig. S4. Evaluation of SNX2112 efficacy on PRCC cells overexpressing AKT1/2 or ERK1/2.**

**
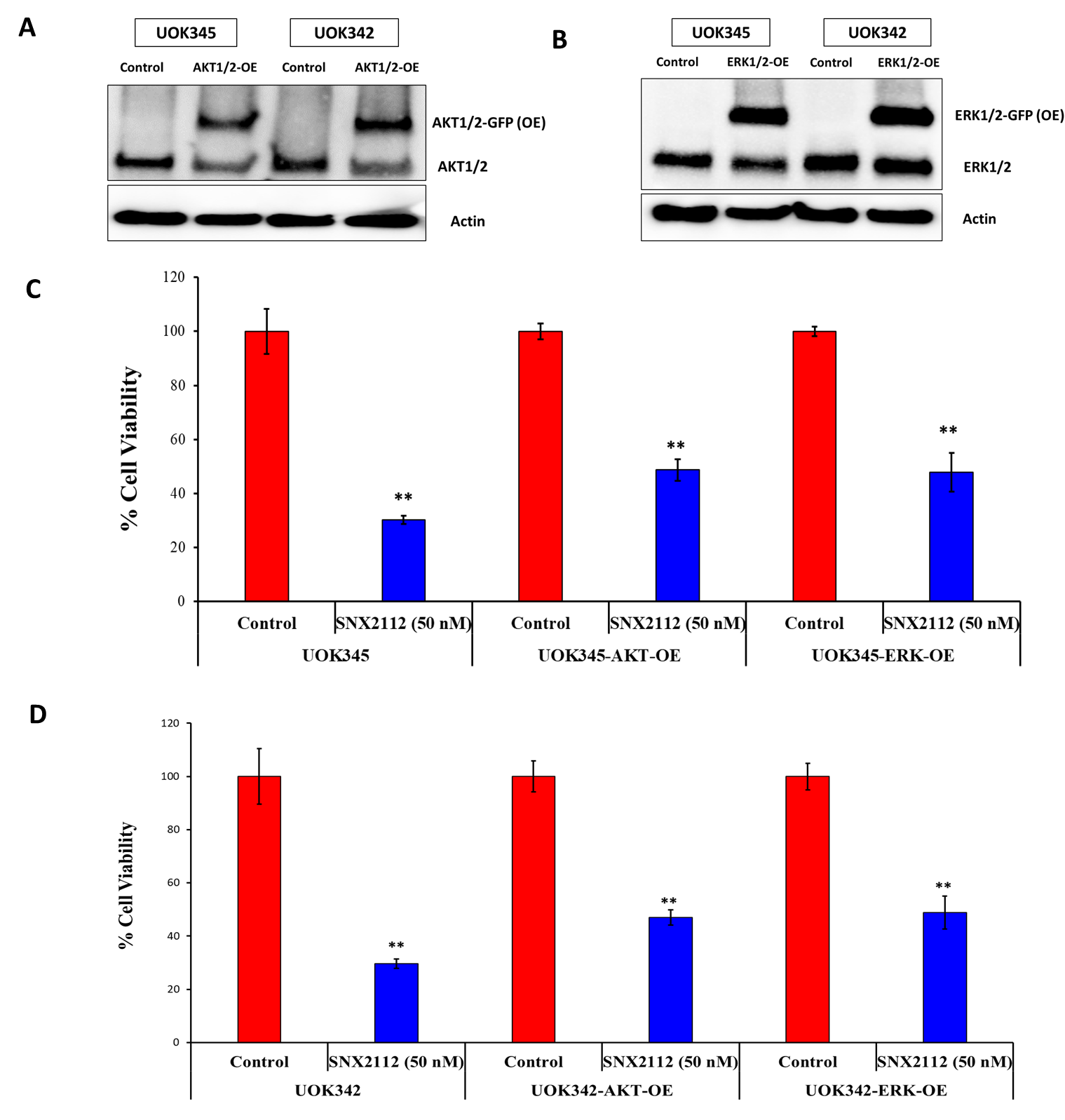
**

**Effect of SNX2112 on PRCC cells overexpressing AKT1/2 or ERK1/2.**

Overexpression of GFP-tagged AKT1/2 (A) or GFP-tagged ERK1/2 (B) using mammalian expression vectors in PRCC cells, UOK345 and UOK342 were confirmed by western blotting. Parent PRCC cells (UOK342, UOK345), PRCC cells overexpressing AKT1/2 (UOK342-AKT-OE and UOK345-AKT-OE), and PRCC cells overexpressing ERK1/2 (UOK342-ERK-OE and UOK345-ERK-OE) were treated with SNX2112 for 72 hours and followed by cell viability assay. Data are representative of three independent experiments. Bar graphs represent the mean ± SD, n=3. ** p<0.00, control vs SNX2112. p< 0.05 is considered significant and was calculated by the two tailed Student’s t test.

**Supplementary Fig. S5. Expression of genes in Papillary Renal Cell Carcinoma TCGA (KIRP) cohort**

**
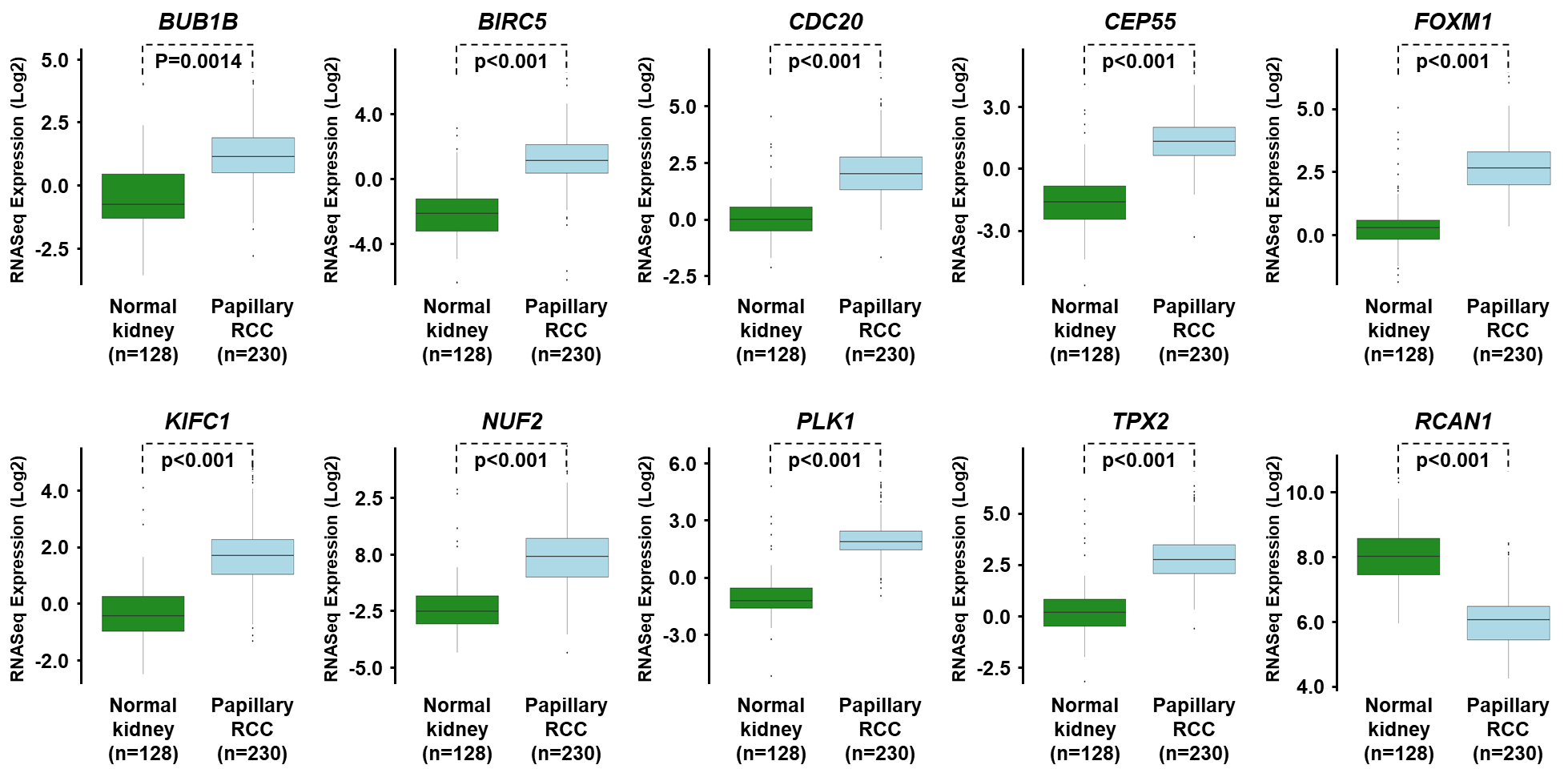
**

**Expression of genes in Papillary Renal Cell Carcinoma TCGA (KIRP) cohort.** The expression of *CDC20, TPX2, CEP55, FOXM1, KIFC1, NUF2, BUB1B, BIRC5* and *PLK1* genes were high and RCAN1 gene, was low in PRCC tumors in comparison to normal controls. p< 0.05 is considered significant and was calculated by the two tailed Student’s t test.

**Supplementary Table S1**

| **TaqMan Assay** | **Catalog Number or Assay ID** | **Manufacture** |
| --- | --- | --- |
| CDC20 TaqMan expression assay | [**Hs00961704_g1**](https://www.thermofisher.com/taqman-gene-expression/product/Hs00961704_g1?CID=&ICID=&subtype=) | Thermo Fisher Scientific |
| FOXM1 TaqMan expression assay | [**Hs01073586_m1**](https://www.thermofisher.com/taqman-gene-expression/product/Hs01073586_m1?CID=&ICID=&subtype=) | Thermo Fisher Scientific |
| BUB1B TaqMan expression assay | [**Hs01084828_m1**](https://www.thermofisher.com/taqman-gene-expression/product/Hs01084828_m1?CID=&ICID=&subtype=) | Thermo Fisher Scientific |
| BIRC5 TaqMan expression assay | [**Hs04985911_s1**](https://www.thermofisher.com/taqman-gene-expression/product/Hs04985911_s1?CID=&ICID=&subtype=) | Thermo Fisher Scientific |
| PLK1 TaqMan expression assay | [**Hs00983227_m1**](https://www.thermofisher.com/taqman-gene-expression/product/Hs00983227_m1?CID=&ICID=&subtype=) | Thermo Fisher Scientific |
| β-Actin TaqMan expression assay | **Hs99999903_m1** | Thermo Fisher Scientific |

List of qRT-PCR TaqMan primer probes (assays) used for gene expression analysis.

**Supplementary Table S2-**

The genetic characterization of PRCC cell lines

| Cell line designation | Patient gender | Patient age | Procurement source | Primary tumor histopathology | MET mutation | Chr. 7 gain | Copies of MET | CDKN2A loss | Additional mutations |
| --- | --- | --- | --- | --- | --- | --- | --- | --- | --- |
| UOK345 | Male | 56 | Pleural fluid | Type 1 PRCC (HPRC) | p.H1112R germline | +1 | +1 | -/- | CUL3 |
| UOK337 | Male | 61 | Abdominal fluid | Type 1 PRCC | p.H1106Q  somatic | +2* | +7 | -/- | - |
| UOK342 | Male | 63 | Ascites | Type 1 PRCC | - | +2* | +6 | -/- | NF2/KRAS |
| UOK332 | Male | 45 | Peritoneal fluid | Type 1 PRCC | - | +1 | +1 | -/+ | BAP1 |

* Additional partial gain(s) of Chr. 7 with *MET* gene; -/- homozygous loss of *CDKN2A*; -/+ heterozygous loss of *CDKN2A. This table is documented using previous study by Yang etal (21)*

**Supplementary Table S3-** file. 1.xls

Differentially expressed genes after SNX2112 treatment in UOK345 and UOK 342 cell lines by RNA seq

**Supplementary Table S4-** file. 2.xls

Gene set enrichment analysis (GSEA) analysis

**Supplementary Table S5:** Relative gene expression in normal (RPTEC) and PRCC cell lines (UOK345, UOK342) by RNA sequencing

**
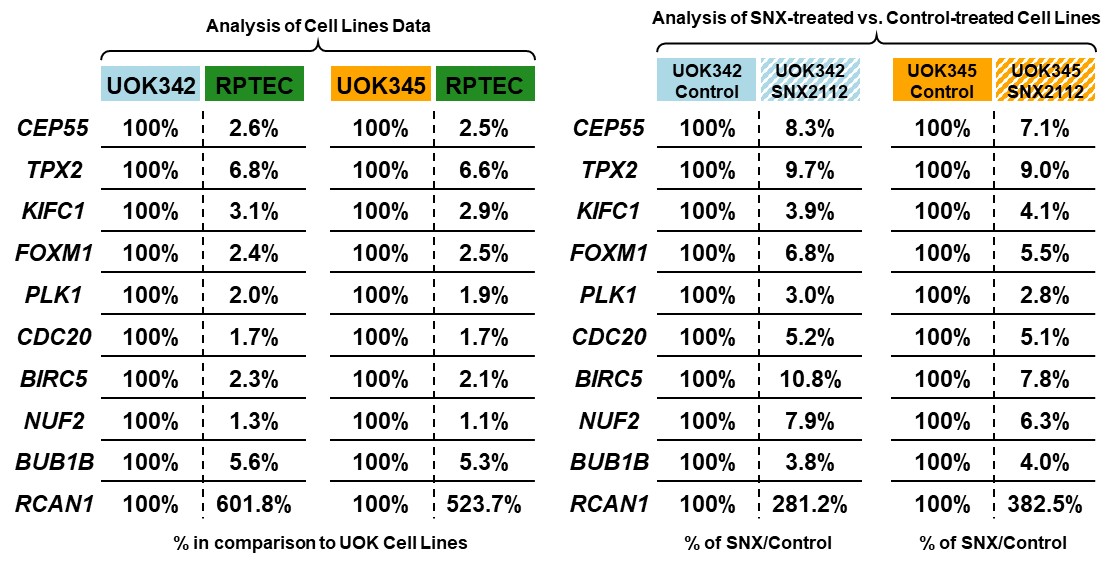
**

Comparative analysis of the RNASeq data from two different experiments (baseline and SNX2112 treated) to evaluate the relative expression levels of selected genes of interest.
